# Predicting tigecycline-related adverse events in infected patients: a machine learning approach with clinical interpretability

**DOI:** 10.3389/fphar.2025.1697929

**Published:** 2025-11-18

**Authors:** Shiya Wu, Yuheng Chen, Wenjie Fan, Xirong Wu, Chaofeng Zhang, Yucang Lin, Qi Lin

**Affiliations:** 1 School of Pharmacy, Fujian Medical University, Fuzhou, China; 2 Department of Pharmacy, The Affiliated Hospital of Putian University, Putian, China; 3 School of Basic Medicine, Putian University, Putian, China; 4 School of Environmental and Biological Engineering, Putian University, Putian, China; 5 Department of Rheumatology and Immunology, The Affiliated Hospital of Putian University, Putian, China; 6 Department of Information, The Affiliated Hospital of Putian University, Putian, China

**Keywords:** tigecycline, machine learning, liver injury, coagulation disorders, risk prediction models

## Abstract

**Background:**

Tigecycline (TGC), while effective against multidrug-resistant infections, is limited by hepatotoxicity and coagulation disorders, yet lacks robust predictive tools.

**Methods:**

We developed an online dynamic nomogram to assess these adverse events using retrospective data from 2,553 TGC-treated patients (2020–2025). Seventy-seven clinical features were analyzed using Boruta and the Least Absolute Shrinkage and Selection Operator (LASSO) for feature selection. Seven machine learning (ML) models were evaluated via ten-fold cross-validation, as well as Receiver Operating Characteristic (ROC) curve and calibration curves, with SHapley Additive exPlanations (SHAP) analysis for interpretability and an online dynamic nomogram for clinical translation.

**Results:**

Logistic regression (LR) outperformed other algorithms, achieving Area Under the ROC Curve (AUC) values of 0.800 (95% CI: 0.727–0.874) for hepatotoxicity and 0.755 (95% CI: 0.665–0.845) for coagulation dysfunction. Independent risk factors for liver injury included prolonged treatment duration, high dosage, ICU admission, hepatitis B virus (HBV) infection, and elevated baseline levels of lactate dehydrogenase (LDH) and gamma-glutamyl transferase (GGT). Risk factors for coagulation dysfunction included extended treatment duration, ICU admission, elevated baseline creatinine (Cr), sepsis, and septic shock. Notably, co-administration of meloxicillin and higher baseline red blood cell (RBC) levels appeared to be protective.

**Conclusion:**

This study constructed an online dynamic nomogram with good discrimination and calibration, which can help to identify high-risk patients and assist clinicians in early risk stratification and individualized treatment planning.

## 1 Introduction

Tigecycline (TGC), a novel glycylcycline-class antimicrobial agent, has demonstrated considerable clinical potential due to its broad-spectrum activity (Korczak et al., 2024), low resistance rate (Fan et al., 2024), and high efficacy against multidrug-resistant pathogens (Xiong et al., 2023). It is primarily indicated for the treatment of complicated intra-abdominal infections, skin and soft tissue infections, and community-acquired pneumonia (Constance and Suzanne, 2012). However, its widespread use has been accompanied by a notable rise in adverse events, particularly hepatotoxicity and coagulation dysfunction, which have emerged as major barriers to its long-term standardized application. Epidemiological studies have reported that the incidence of TGC-related liver injury ranges from 1.6% to 28.9%, with severe cases potentially progressing to liver failure or death (Shi et al., 2021; Shi et al., 2022; Liu Y. X. et al., 2021; Chen and Shi, 2018). In parallel, numerous reports have demonstrated that TGC impairs coagulation function—manifested by reduced fibrinogen (FIB) levels—thereby increasing the risk of hemorrhage and severe complications (Zhang et al., 2023; Leng et al., 2019; Ma et al., 2024; Guo et al., 2022).

Furthermore, considerable interindividual variability in TGC pharmacokinetics and pharmacodynamics leads to significant differences in therapeutic outcomes and susceptibility to adverse events, complicating its clinical management (Dorn et al., 2018; Ruiz et al., 2020). Thus, balancing the safety and efficacy of TGC has become a critical clinical challenge. Identifying risk factors for adverse outcomes remains a cornerstone of clinical practice and public health (Boukhlal et al., 2024). Previous studies have implicated variables such as baseline alanine aminotransferase (ALT) and albumin abnormalities, ICU admission, and treatment duration as independent risk factors for TGC-related liver injury (Jiang T. et al., 2022; Yu et al., 2022). Zhang et al. further highlighted that patients receiving voriconazole, those with cancer, intra-abdominal infections, or septic shock were at significantly higher risk (Zhang et al., 2025). Similarly, Liu et al. found that age, treatment duration, and baseline FIB levels were associated with hypofibrinogenemia, particularly in patients with hematologic malignancies (Guo et al., 2024; Liu J. et al., 2021). The relationship between high TGC doses and adverse events, however, remains contentious (Geng et al., 2018; Gong et al., 2019). Traditional statistical models used in these studies often overlook individual heterogeneity and the complexity of multifactorial interactions, thereby limiting predictive accuracy and adaptability. In contrast, machine learning (ML) techniques offer distinct advantages in risk prediction by effectively handling high-dimensional datasets and capturing complex, non-linear relationships (Das et al., 2024; Wu et al., 2020).

Globally, ML algorithms have been successfully applied to predict adverse reactions to anticancer and antiviral agents, identify key biomarkers, and optimize therapeutic strategies (Arab et al., 2024; Saoud et al., 2024). Additionally, ML has demonstrated value in enhancing drug safety surveillance (Eujin et al., 2023), forecasting pharmacological updates (Watanabe et al., 2024), and addressing limitations inherent in traditional pharmacovigilance systems. Despite these advancements, studies applying ML to predict TGC-related adverse events remain scarce. To address this gap, our study constructed and validated a risk prediction model for TGC-related hepatotoxicity and coagulation dysfunction using 7 ML algorithms. We further applied the SHapley Additive exPlanations (SHAP) method to interpret the model output as a means of identifying key predictive features. Ultimately, we developed an online prediction webpage that was able to predict both whether an adverse reaction would occur with the use of TGC and, separately, whether TGC would cause liver injury or coagulation dysfunction. This study provides a visual and interpretable basis for clinical decision-making and strongly supports the advancement of personalized TGC dosing strategies.

## 2 Methods

### 2.1 Study population and subgroups

This work was approved by the Ethics Committee of the Affiliated Hospital of Putian University (Approval ID: 2025141), and the research flowchart is shown in Supplementary Figure S1. This study included hospitalized patients treated with TGC at the Affiliated Hospital of Putian University between January 2020 and January 2025. Data from January 2020 to December 2023 were used for model development, while data from January 2024 to January 2025 were used for external validation. The inclusion criteria were as follows: (1) age >18 years; (2) TGC therapy duration >3 days; and (3) availability of complete laboratory data. The exclusion criteria were as follows: (1) incomplete medical records; (2) pre-existing liver injury or coagulation disorders; and (3) pregnancy. Eligible patients were classified into two cohorts: the liver injury risk cohort and the coagulation dysfunction risk cohort. We followed up with patients receiving TGC for 3 months to avoid missing positive events. Each cohort was randomly divided in a 7:3 ratio into training and test sets. Patient demographics and baseline characteristics were collected for further evaluation.

### 2.2 Definition of study outcomes

Drug-induced liver injury (DILI) related to TGC was defined according to established criteria (Edmond et al., 2023), meeting at least one of the following: (1) ALT ≥5×ULN; (2) alkaline phosphatase (ALP) ≥ 2×ULN (especially when accompanied by elevated gamma-glutamyl transferase (GGT) and after exclusion of bone pathology); (3) ALT ≥3×ULN and total bilirubin (TBIL) ≥ 2×ULN. Liver injury was further subclassified into hepatocellular, cholestatic, or mixed types and graded for severity (levels 1–4) according to international guidelines (Yu et al., 2017). The Roussel Uclaf Causality Assessment Method (RUCAM) was employed to evaluate the causal relationship between TGC and liver injury, with a score ≥6 considered indicative (Supplementary Table S1) (Ciricillo et al., 2024).

Coagulation dysfunction was diagnosed during TGC treatment if any of the following criteria were met: (1) FIB <2 g/L; (2) international normalized ratio (INR) outside the standard range (0.8–1.2); (3) prothrombin time (PT) prolonged by > 3 s beyond the standard range (9–13 s); (4) activated partial thromboplastin time (APTT) prolonged by > 10 s beyond standard range (20–40 s); (5) platelet count (PLT) outside the standard range (125 × 10^9^ L^-1^–350 × 10^9^ L^-1^).

Additionally, the clinical efficacy of TGC treatment was categorized as cured, improved, or ineffective. Detailed diagnostic criteria are available in the Supplementary File.

### 2.3 Feature selection

To ensure robust variable selection, a combined approach using Least Absolute Shrinkage and Selection Operator (LASSO) regression and the Boruta algorithm was adopted. Initially, univariate analyses were conducted on the training set to establish a preliminary feature pool, incorporating clinically relevant variables reported in prior literature to be associated with TGC-related hepatotoxicity and coagulation abnormalities. Variables were then categorized into categorical or continuous variables based on clinical features. Categorical variables were coded as dummy variables, whereas continuous variables were standardized using a Z-score with a mean of 0 and a standard deviation of 1. Multicollinearity was assessed by variance inflation factor (VIF) analysis, and features exceeding a VIF >5 were excluded from subsequent analyses. LASSO regression was then applied with a binomial family specification and alpha set to 1 to enforce pure LASSO regularization. The penalty parameter λ was optimized through ten-fold cross-validation, and variable selection was based on both lambda. min and lambda.1se to retain the most informative features (Liu et al., 2023). Simultaneously, the Boruta algorithm was employed to assess feature importance by comparing original variables against randomized “shadow” features across 500 iterations or until stability was achieved (Sun et al., 2024). The intersection of both methods yielded a high-confidence feature subset, which was used to construct robust ML models. Analysis was conducted using R programming (version 4.4.0).

### 2.4 Model development, evaluation and interpretation

Seven ML algorithms were used for model construction: Logistic Regression (LR), Decision Tree (DT), K-Nearest Neighbors (KNN), Extreme Gradient Boosting (XGBoost), Light Gradient Boosting Machine (LightGBM), Random Forest (RF), and Gaussian Naive Bayes (GNB). Ten-fold cross-validation was performed for model training and optimization. Hyperparameters for each algorithm were fine-tuned using a grid search strategy.

Model performance was assessed using multiple evaluation metrics, including the Receiver Operating Characteristic (ROC) curve and Brier score. A lower Brier score indicates superior model calibration and discrimination (Angraal et al., 2020). To enhance model interpretability, SHAP was used to quantify the contribution of each feature at both global and individual levels (Jiang C. et al., 2022). Additionally, a dynamic nomogram was developed to provide a user-friendly visualization of individual risk predictions and facilitate clinical decision-making. Specific model parameters are detailed in the Supplementary Parameters. Analyses were conducted using R programming (version 4.4.0) and Python (version 3.7).

### 2.5 Validation and updating of a risk prediction model

The Kolmogorov-Smirnov (K-S) test was used to verify the distributional consistency of key features between the development and validation datasets. After confirming compatibility, the validation dataset was applied to the original model for external validation. Model performance was further evaluated using the area under the ROC curve (AUC) and calibration plots. To update the model, the development and validation datasets were merged and used to retrain the model. The Wilcoxon rank-sum test was then applied to compare predictive outputs from the original and updated models. A p-value >0.05 indicated no significant difference, suggesting the original model remained adequate, whereas a p-value <0.05 suggested that model adjustment was warranted.

### 2.6 Statistical analysis

Missing values (<20% for all variables; Supplementary Tables S2, 3) were addressed using multiple imputation in SPSS (version 27.0). Imputation was performed using the MICE procedure with predictive mean matching, set to 5 imputations and a maximum of 50 iterations. The K-S test was used to assess normality of continuous variables. Normally distributed data were expressed as mean ± standard deviation, while skewed data were reported as medians with interquartile ranges. Between-group comparisons were conducted using Student’s t-test for normally distributed data and Mann-Whitney U test for non-normally distributed data. Categorical variables were presented as counts and percentages, and compared using the chi-square test. A p-value <0.05 was considered statistically significant.

## 3 Results

### 3.1 Baseline characteristics of the study population

During the model development phase, we initially screened 2,006 hospitalized patients who received TGC, collecting data on 77 clinical variables, including demographics, laboratory tests, and medication records (e.g., age, gender, ICU admission, treatment duration, concomitant medications, and inflammatory markers). Detailed metrics are shown in Supplementary Tables S4. Based on predefined inclusion and exclusion criteria, 1,073 cases were included in the liver injury risk cohort and 612 in the coagulation dysfunction cohort. Each cohort was randomly divided into training and test sets in a 7:3 ratio, with no significant differences in baseline characteristics between the groups (Supplementary Tables S5, 6; all P > 0.05). For external validation, 547 patients were screened, of whom 380 were retained for liver injury risk analysis and 114 for coagulation dysfunction assessment after eligibility filtering.

### 3.2 Feature selection and clinical profiling of TGC-Induced hepatotoxicity

**FIGURE 1:**
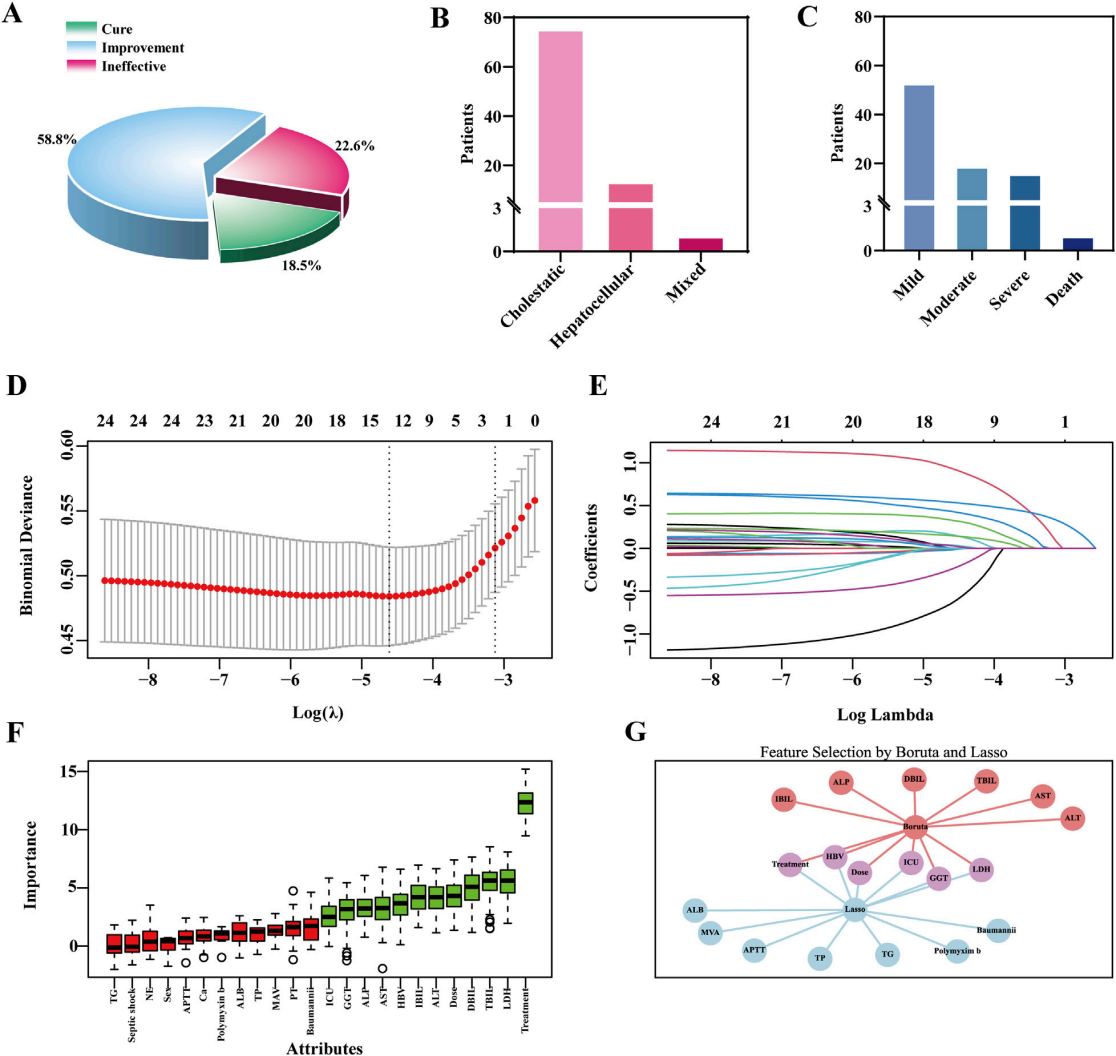
Clinical outcomes of TGC-related Liver Injury and feature selection using the LASSO and boruta algorithm. **(A)** Clinical efficacy outcomes of TGC treatment. **(B)** Types of liver injury caused by TGC. **(C)** Severity grading of TGC-related liver injury. **(D)** Feature selection using the LASSO regression model. **(E)** Coefficient trajectories of variables in the LASSO regression model. **(F)** Important predictors identified by the Boruta algorithm. **(G)** Overlapping predictors identified by both LASSO and Boruta algorithms.

Among patients with TGC-related liver injury (Supplementary Tables S7), 89.9% had a highly probable causality rating according to the RUCAM scale, with an overall incidence of 8.30%. Liver function test abnormalities were common: 43.1% exhibited elevated ALP levels, and 35.5% had increased direct bilirubin (DBIL), indicating notable hepatic involvement. While TGC treatment led to clinical improvement in 58.8% of affected patients, the cure rate was only 18.5%, and 22.6% experienced no improvement (Figure 1A). Most liver injuries were classified as mild and cholestatic in nature. However, one fatal case was recorded (Figures 1B,C), underscoring the importance of vigilant liver function monitoring during TGC therapy.

To identify predictors of TGC-related hepatotoxicity, univariate analysis was conducted on the training set (Supplementary Tables S8), identifying 14 variables with potential associations (P < 0.1), including treatment duration and maintenance dose. These were combined with 24 clinically relevant features, such as sex and ALT levels, previously reported in the literature (Alraish et al., 2020; Fan et al., 2020; Shi et al., 2021), and subjected to LASSO regression for dimensionality reduction. Ten-fold cross-validation identified the optimal penalty parameter (λ = 0.0099) (Figure 1D). At this λ value, 13 features had non-zero coefficients (Figure 1E), indicating their relevance to the model. In parallel, the Boruta algorithm identified 12 important predictors (Figure 1F). The VIF values for all features were below the threshold of 5, indicating no substantial multicollinearity among the variables (Supplementary Figures S2A,B; Supplementary Tables S9). By integrating the outputs from both LASSO and Boruta, key features were finalized for model construction, including treatment duration, maintenance dose, hepatitis B virus (HBV) infection status, ICU admission, GGT, and lactate dehydrogenase (LDH) (Figure 1G).

### 3.3 Performance and interpretability of hepatotoxicity prediction models

**FIGURE 2:**
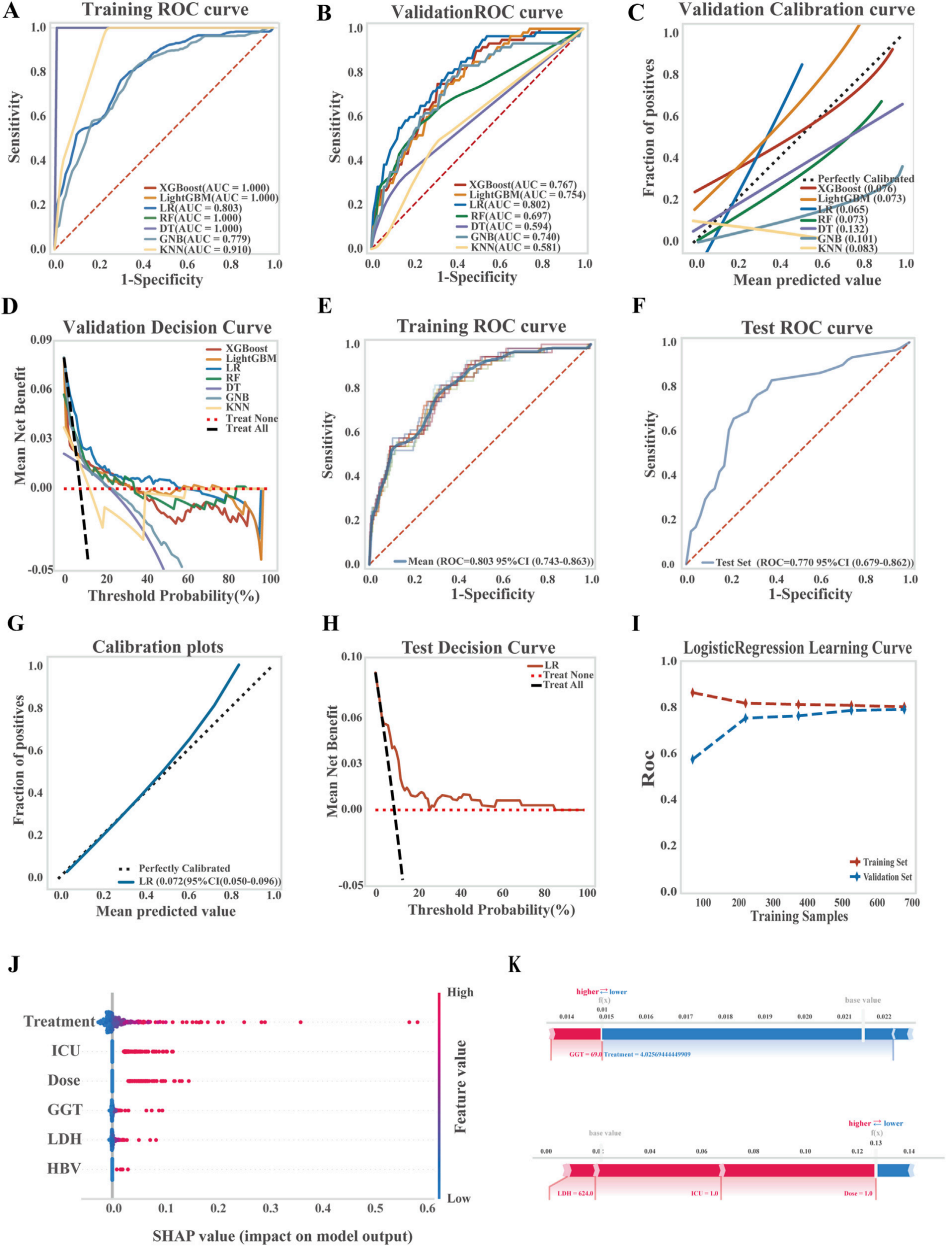
Development, performance comparison, and interpretability analysis of ML models for liver injury prediction. **(A)** ROC curve of the training set for all models; **(B)** ROC curve of the validation set for all models; **(C)** Calibration curve comparing predicted and observed outcomes; **(D)** Decision curve analysis (DCA) for the validation set; **(E)** ROC curve of the LR model in the training set; **(F)** ROC curve of the LR model in the test set; **(G)** Calibration curve of the LR model; **(H)** DCA curve of the LR model in the test set; **(I)** Learning curve of the LR model; **(J)** SHAP summary plot (dendrogram) showing feature importance in the LR model; **(K)** SHAP-based interpretability analysis for two independent samples, illustrating each feature’s contribution to risk prediction.

Seven ML algorithms were applied to the liver injury risk dataset using ten-fold cross-validation. Model performance was evaluated through ROC and PR curves, AUC, accuracy, precision, sensitivity, and positive predictive value. Among them, the LR model consistently outperformed others across key evaluation metrics (Figures 2A–D; Supplementary Figure 3).

The LR model demonstrated strong discriminatory power, achieving an average AUC of 0.803 (95% CI: 0.743–0.863) on the training set and 0.770 (95% CI: 0.679–0.862) on the test set. A Brier score of 0.072 (95% CI: 0.050–0.096) reflected good calibration and predictive accuracy. Decision curve analysis confirmed the clinical utility of the model, showing a net benefit over both treat-all and treat-none strategies across threshold probabilities from 18% to 97%. Learning curves demonstrated stable model performance with increasing sample size, with no evidence of overfitting or underfitting, indicating successful model training and generalizability (Figures 2E–I; see Supplementary Tables S10 for the LR equation).

To explore model interpretability, SHAP analysis was performed to identify and rank key predictors of TGC-related hepatotoxicity. The most influential features included treatment duration, ICU admission, maintenance dose, baseline GGT, LDH, and HBV infection, as visualized by sample SHAP values (Figure 2J). Figure 2K presents two representative patient cases, highlighting the contribution of each feature to the model’s predictions. Red bars indicate features that increase risk, while blue bars indicate protective factors.

### 3.4 Feature selection and clinical profiling of TGC-Induced coagulation disorder

**FIGURE 3:**
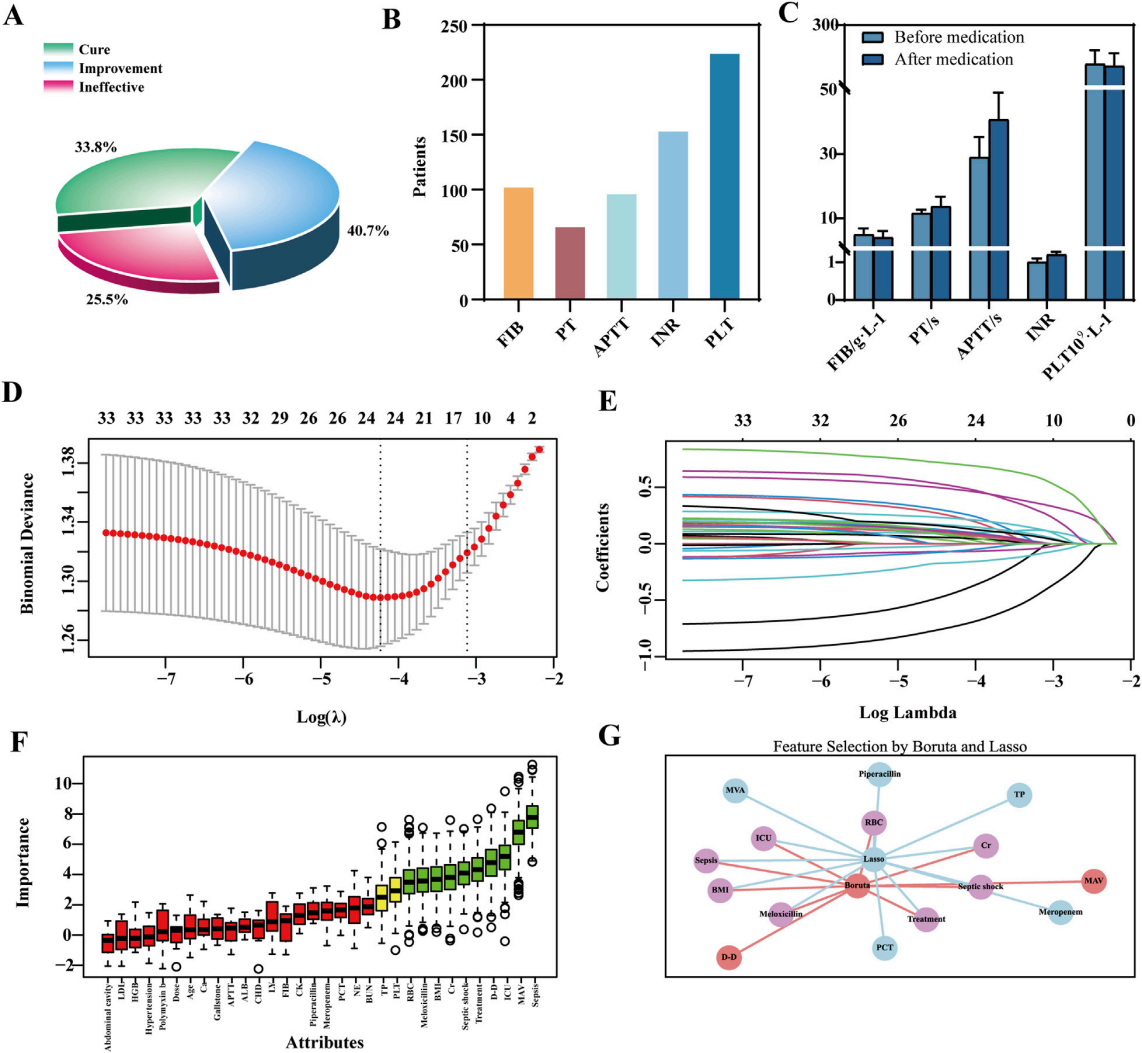
Clinical outcomes of TGC-induced coagulation disorders and feature selection via LASSO-Boruta Algorithm. **(A)** Clinical efficacy of TGC treatment; **(B)** Types of coagulation dysfunction associated with TGC; **(C)** Changes in coagulation indices before and after TGC administration; **(D)** Feature selection using the LASSO regression model; **(E)** Variable coefficient trajectories in the LASSO regression model; **(F)** Key predictors identified by the Boruta algorithm; **(G)** Overlapping predictors identified by both LASSO and Boruta methods.

Among the 612 TGC-treated patients, 317 (51.8%) developed coagulopathy. Efficacy analysis revealed symptom improvement in 40.7% of cases, a cure rate of 33.8%, and a non-response rate of 25.5%. Coagulation dysfunction primarily manifested as abnormalities in PLT, INR, and FIB levels. TGC treatment resulted in prolonged PT and APTT, elevated INR, and decreased PLT and FIB levels (Figures 3A–C).

Univariate analysis identified 27 significant predictors of coagulation dysfunction, including body mass index (BMI) and HBV status (Supplementary Tables S11). These, along with 33 additional indicators such as gender and baseline ALB reported in previous studies (Firat et al., 2024; Hu et al., 2020; Ma et al., 2024), were included in the LASSO regression for dimensionality reduction. The optimal model performance was achieved at λ = 0.0444, yielding 13 features with non-zero coefficients (Figures 3D,E). The Boruta algorithm identified 10 key predictors (Figure 3F). The VIF values for all features were below the threshold of 5, indicating no substantial multicollinearity among the variables (Supplementary Figures S2C,D, Supplementary Tables S12). By comparing the LASSO and Boruta results, a common subset of features was selected for model construction, including treatment regimen, ICU admission, septic shock, sepsis, co-administration of meloxicillin-sulbactam, Cr, red blood cell (RBC) count, and BMI (Figure 3G).

### 3.5 Performance and interpretability of coagulation disorders models

**FIGURE 4:**
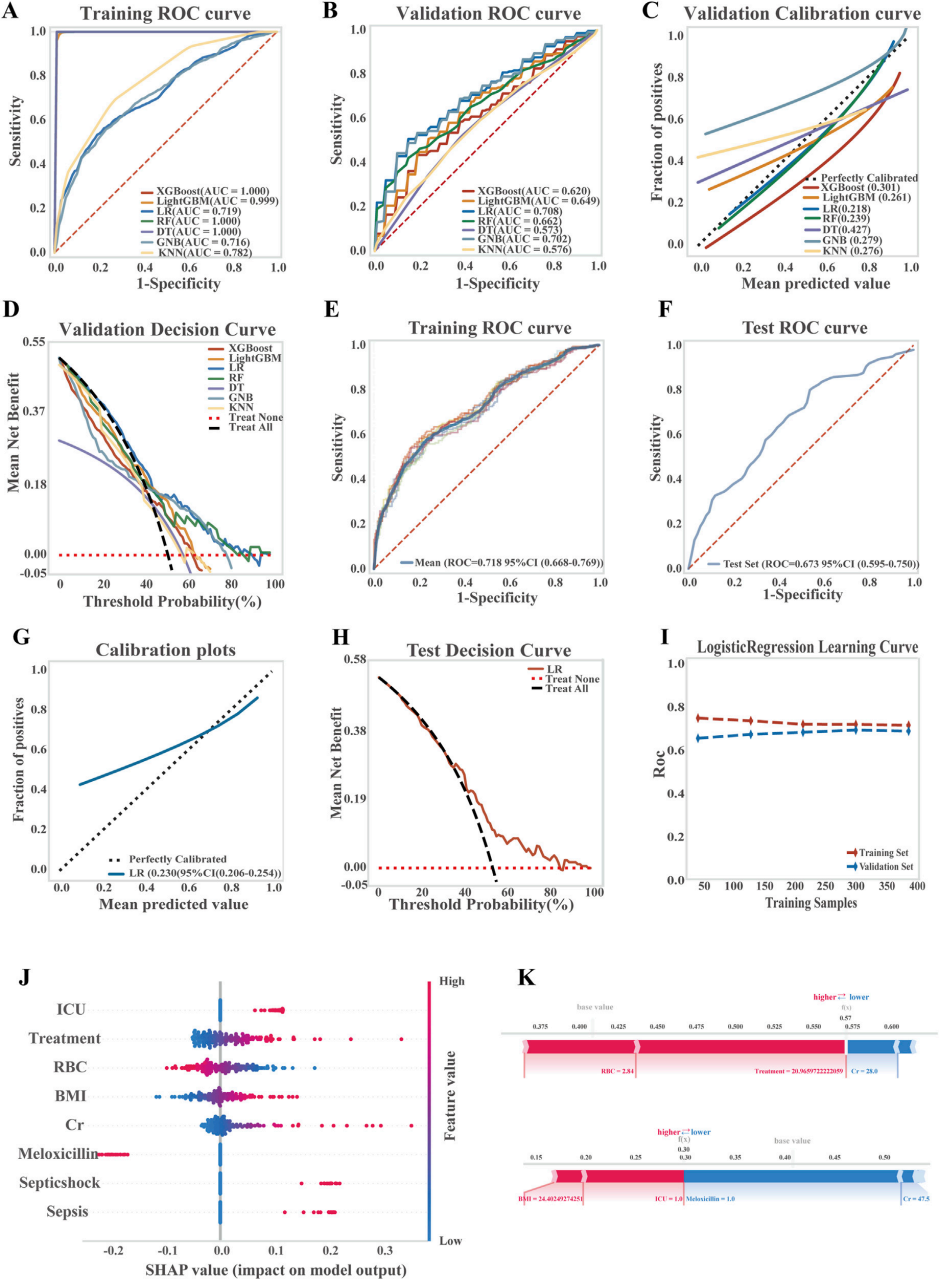
Development, performance comparison, and interpretability analysis of ML models for predicting TGC-induced coagulation disorders. **(A)** ROC curve of the training set for all models; **(B)** ROC curve of the validation set for all models; **(C)** Calibration curve for overall model performance; **(D)** Decision curve analysis (DCA) of the validation set; **(E)** ROC curve of the training set for the LR model; **(F)** ROC curve of the test set for the LR model; **(G)** Calibration curve of the LR model; **(H)** DCA of the LR model on the test set; **(I)** Learning curve of the LR model; **(J)** SHAP summary plot showing feature importance in the LR model; **(K)** SHAP-based interpretability analysis of two representative patient samples.

Seven ML algorithms were systematically evaluated for predicting the risk of TGC-related coagulopathy. Model performance was assessed using ROC and calibration curves, AUC forest plots, and evaluation metrics such as PR curves, accuracy, and precision (Figures 4A–D; Supplementary Figure 4). Among all models, LR demonstrated the best overall performance.

In the LR model, moderate discriminative ability was observed, with an average AUC of 0.718 (95% CI: 0.668–0.769) for the training set and 0.673 (95% CI: 0.595–0.750) for the test set. A Brier score of 0.230 (95% CI: 0.206–0.254) suggested room for further improvement in prediction accuracy. Decision curve analysis (DCA) indicated a clear net benefit of the model over both treat-all and treat-none strategies across a threshold probability range of 38%–90%, demonstrating its clinical applicability. Learning curves confirmed progressive and stable model performance with increasing data volume, without signs of overfitting or underfitting, thereby validating the model’s robustness (Figures 4E–I; LR equation is provided in Supplementary Tables S13).

To interpret the model’s predictions, key predictors of TGC-associated coagulopathy were evaluated using SHAP values. As shown in Figure 4J, features were ranked by importance, with red indicating a higher feature value and blue a lower one. The most influential risk factors, in descending order, included ICU admission, treatment duration, and others, while baseline RBC levels and co-administration of meloxicillin-sulbactam were identified as protective factors. Additionally, SHAP-based interpretability analysis of two representative patient samples (Figure 4K) illustrated how individual features contributed to the predicted risk scores.

### 3.6 Periodic validation and updating of risk prediction models

**FIGURE 5:**
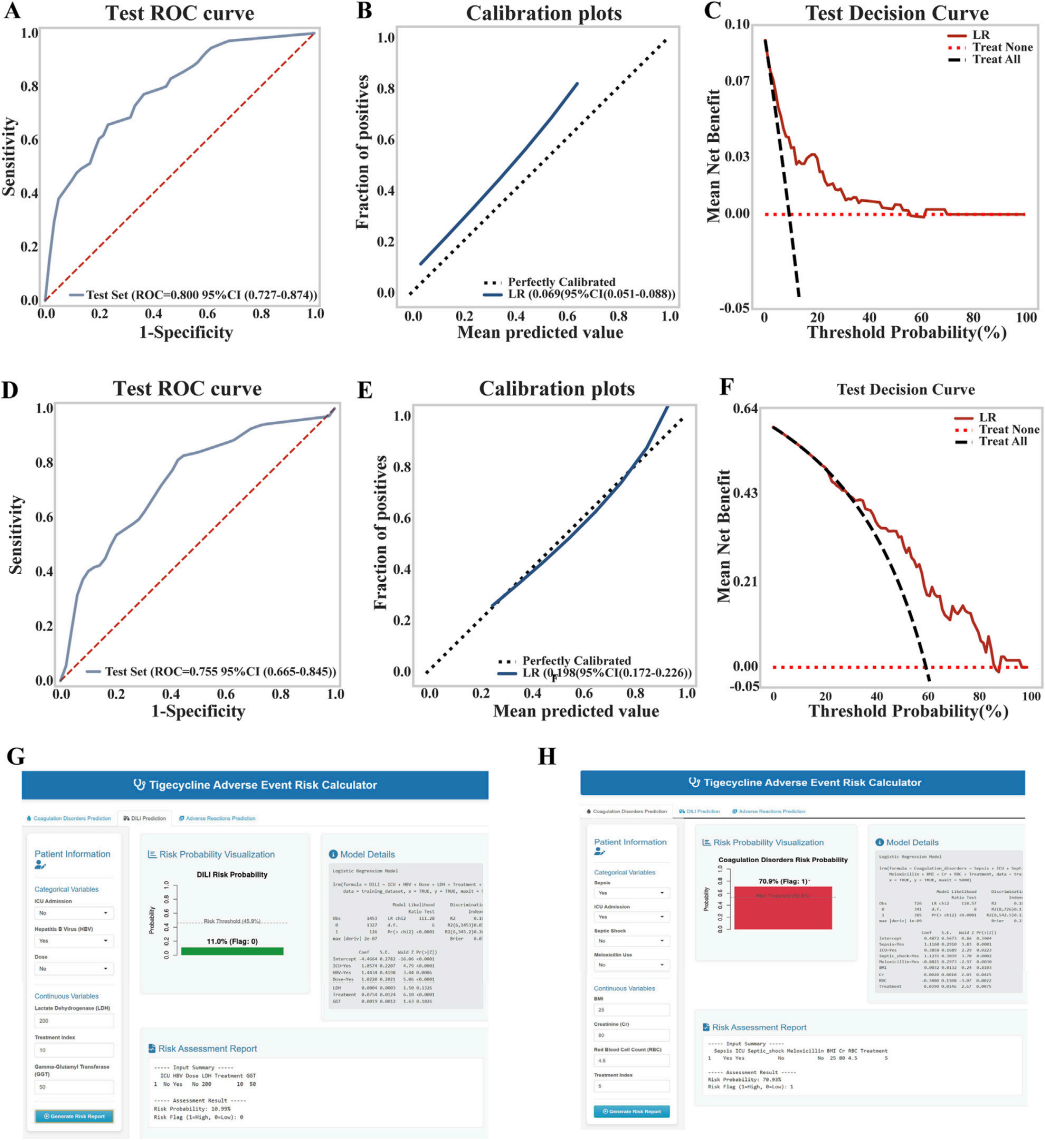
Updates to the LR model for liver injury and coagulation dysfunction. **(A)** ROC curve of the liver injury model on the test set; **(B)** Calibration curve of the liver injury model; **(C)** Decision curve analysis (DCA) of the liver injury model on the test set; **(D)** ROC curve of the coagulation dysfunction model on the test set; **(E)** Calibration curve of the coagulation dysfunction model; **(F)** DCA of the coagulation dysfunction model on the test set; **(G)** Dynamic nomogram of the updated liver injury model; **(H)** Dynamic nomogram of the updated coagulation dysfunction model.

In the time-series validation of the liver injury prediction model (n = 380), the new test set demonstrated strong discriminative ability, with an AUC of 0.800 (95% CI: 0.727–0.874) and a Brier score of 0.069, indicating excellent alignment between predicted and actual outcomes. The model provided a higher net clinical benefit compared to both full intervention and no intervention strategies within a threshold probability range of 18%–70% (Figures 5A–C). Overall, the model’s performance remained robust. After reconstruction using the combined training and validation datasets, no significant difference was observed between the original and updated models (p = 0.243). Consequently, the updated model was adopted, and a dynamic nomogram was generated for clinical implementation (Figure 5G). Similarly, in the temporal validation of the coagulation dysfunction prediction model (n = 114), the new test set demonstrated improved predictive performance, with an AUC of 0.755 and a lower Brier score of 0.198, indicating enhanced predictive accuracy and clinical utility within the 38%–90% threshold probability range (Figures 5D–F). Similar to the liver injury model, no significant performance difference was found between the original and updated versions (p = 0.757), leading to the adoption of the revised model for the final clinically applicable version (Figure 5H). The URL for the prediction model is https://tigecyclineriskprediction.shinyapps.io/Shiny/.

## 4 Discussion

The present study systematically identified six independent risk factors for TGC-related hepatotoxicity, not only validating established risk parameters but also introducing novel predictors, thereby advancing the current understanding of TGC safety profiles. Notably, a maintenance dose exceeding 100 mg/day was associated with a 2.6-fold increased risk of liver injury (95% CI: 2.391–5.546, p < 0.001), corroborating previous findings that highlighted the heightened risk associated with high-dose TGC regimens (Bai et al., 2023; Ruiz et al., 2020). A potential mechanism may involve TGC-related inhibition of mitochondrial respiratory chain complex II, leading to impaired ATP synthesis in hepatocytes (Chen et al., 2024), although the precise dose‐response relationship remains controversial (Falagas et al., 2014; Yu et al., 2022).

In addition to dosage, treatment duration emerged as a critical determinant of hepatotoxicity risk. Our analysis identified TGC administration for >12 days as an independent risk factor, consistent with prior studies that reported risk thresholds at >8 days (Jiang T. et al., 2022), and >14 days (Shi et al., 2021). The underlying pathophysiology may involve TGC-related mitochondrial oxidative stress, activation of nuclear factor-κB signaling pathways, and upregulation of pro-inflammatory cytokines (IL-1β, IL-6, TNF-α), which exacerbate hepatocellular injury (Koch et al., 2023). These findings support enhanced hepatic monitoring and regular benefit-risk reassessment for patients receiving TGC beyond 7 days to guide treatment decisions. The establishment of dose- and duration-dependent risk thresholds provides actionable guidance to optimize TGC therapy while mitigating hepatotoxicity.

Beyond pharmacological parameters, our study identifies HBV infection as a significant risk factor for TGC-related hepatotoxicity—a finding that warrants further mechanistic investigation. Earlier investigations, while acknowledging the potential role of pre-existing liver conditions, failed to establish a clear link with HBV, possibly because relevant studies were underpowered or examined heterogeneous liver diseases as a collective entity. Our findings resolve this ambiguity by pinpointing HBV infection as an independent risk factor through robust multivariate analysis in a sizable cohort. In HBV-infected individuals, the pre-existing state of immune activation and persistent hepatic inflammation is hypothesized to lower the threshold for drug-induced liver injury. This could create a synergistic effect, whereby the baseline inflammatory milieu exacerbates TGC-induced hepatotoxicity. However, the precise immunopathological interplay underlying this potential synergy remains unclear and requires direct validation through future studies.

Consistent with previous reports, a patient’s status as critically ill, necessitating ICU admission, was significantly associated with hepatotoxicity. This association is likely not attributable to the ICU setting itself, but rather serves as a marker of heightened risk due to the severe underlying pathophysiology. The collective burden of systemic inflammation, hepatic hypoperfusion, frequent polypharmacy with potential drug interactions, and associated metabolic disturbances in critically ill patients may synergistically impair liver function and drug clearance, thereby increasing susceptibility to TGC-related liver injury (Yu et al., 2022).

To our knowledge, this study is the first to propose that elevated baseline GGT and LDH levels may serve as early warning biomarkers for TGC-related hepatotoxicity. GGT, a well-established marker of hepatobiliary dysfunction, has demonstrated prognostic value in DILI, particularly in isoniazid toxicity (Balkrishna et al., 2024). Similarly, LDH has proven valuable in pharmacotoxicology, where elevated serum levels correlate with hepatocellular damage in acetaminophen overdose (Liu et al., 2022) and sepsis severity (Akin et al., 2025). Although the association between GGT/LDH and TGC-related hepatotoxicity remains to be fully elucidated, TGC is known to cause hepatocellular injury via mitochondrial dysfunction and oxidative stress (Tan et al., 2017). We hypothesize that elevated baseline GGT reflects compromised oxidative stress defenses, rendering hepatocytes more vulnerable to TGC toxicity, while increased LDH may indicate disruptions in energy metabolism that amplify mitochondrial damage. These insights open new research avenues for early biomarkers and mechanisms of TGC hepatotoxicity. Large-scale clinical studies are essential to validate the predictive utility of GGT and LDH and to define clinically meaningful thresholds.

While hepatotoxicity is a major concern, our findings also emphasize the risk of TGC-related coagulation disorders. First, prolonged treatment duration was a significant risk factor for coagulopathy. Previous studies have shown that extended TGC use impairs vitamin K epoxide reductase activity, thereby reducing synthesis of coagulation factors and contributing to hypofibrinogenemia. Second, the status of patients admitted to the ICU was significantly associated with coagulation disorders. We believe that this association stems primarily from the critical pathophysiologic state signified by ICU admission, where inherent risk factors (e.g., sepsis, shock, and systemic inflammation) are the primary drivers of coagulation disorders.

We further identified, for the first time, that a BMI >21.98 kg/m^2^ is an independent risk factor for TGC-related coagulopathy (OR = 1.125, 95% CI: 0.958–1.324). This may be attributed to obesity-associated chronic inflammation, which promotes activation of the coagulation system, increases hepatic metabolic burden, and disrupts gut microbiota, potentially impairing vitamin K-dependent coagulation factor activation (Zhang et al., 2021). Additionally, elevated baseline serum Cr levels in patients with TGC-related coagulopathy suggest impaired renal function leads to reduced TGC clearance, drug accumulation, and greater anticoagulant effects. These findings underscore the importance of intensive coagulation monitoring in patients with renal insufficiency receiving TGC.

We also discovered that systemic inflammatory conditions significantly increase the risk of coagulation. Specifically, septic shock (OR = 3.065) and sepsis (OR = 3.015) were identified as strong independent predictors of TGC-related coagulopathy. These conditions are typically associated with multi-organ dysfunction and robust inflammatory responses. Pathogen-associated molecular patterns stimulate monocytes and macrophages to release tissue factor, triggering the extrinsic coagulation cascade. Simultaneously, inflammation inhibits anticoagulant pathways, increasing thrombin generation, fibrin deposition, and microthrombus formation, all of which contribute to coagulopathy. Thus, close monitoring of coagulation parameters is crucial in patients with septic shock or sepsis receiving TGC.

Moreover, our study revealed that co-administration of TGC with meloxicillin was associated with a significantly reduced risk of coagulation dysfunction (OR = 0.630, 95% CI: 0.520–0.780). An alternative explanation for this protective effect, beyond direct pharmacological antagonism of TGC toxicity, is that meloxicillin, by effectively controlling the primary infection, reduces sepsis severity—a key driver of coagulation dysfunction. This indirect mechanism is supported by known synergism between TGC and β-lactams against resistant pathogens such as MRSA (Aktas, 2021), which enhances bacterial clearance. Concurrently, meloxicillin’s spectrum of activity may help preserve gut microbiota and vitamin K production, further protecting against coagulopathy. Thus, the net benefit likely stems from combined antimicrobial efficacy and microbiome preservation, underscoring the clinical utility of this regimen.

Concurrently, we identified elevated baseline RBC levels as a protective factor against TGC-related coagulopathy. This may be due to improved hemorheology, enhanced microcirculatory perfusion, balanced coagulation-fibrinolysis activity, and better oxidative stress resistance (Nasralddin et al., 2025). These findings suggest the need for careful coagulation monitoring in anemic patients receiving TGC and highlight the potential for exploring erythrocyte-mediated protective mechanisms in drug-related coagulopathies.

While the discriminative performance of our hepatotoxicity model (AUC: 0.800) is consistent with prior work by Zhang et al. (AUC: 0.800–0.820), the present study provides several crucial advancements. Our research benefits from a substantially larger sample size (n = 2,553 vs. n = 357), lending greater robustness to the identified risk factors. Furthermore, we expanded the predictive landscape by identifying baseline GGT and LDH as novel biomarkers and, for the first time, developed a dedicated predictive model for TGC-associated coagulation dysfunction, thereby addressing significant gaps in the existing evidence base. Collectively, these findings provide a more comprehensive safety framework for TGC use, enabling improved risk stratification and personalized patient monitoring in clinical practice.

Also, this study had some limitations needed to further explorer. First, the model was developed from a hospital sample of 2,553 TGC-treated patients over a 5-year period, but its generalizability might be influenced by the unique local patient demographics and prescribing culture. Second, as our model has not yet been validated in large external cohorts, further multicenter studies will be required to verify its broader applicability and accuracy.

## 5 Conclusion

We developed and validated an interpretable ML model to identify risk and protective factors associated with TGC-related liver injury and coagulation dysfunction. A comprehensive comparison of 7 ML algorithms revealed that the LR model achieved the best performance in both training and test datasets (liver injury model test set AUC: 0.801, Brier score: 0.075; coagulation dysfunction model test set AUC: 0.755, Brier score: 0.198. Key risk factors were identified, and model interpretability was enhanced using SHAP analysis. The resulting dynamic nomograms offer direct support for clinical decision-making and represent a valuable tool for individualized TGC dosing strategies.

## Data Availability

The original contributions presented in the study are included in the article/Supplementary Material. Further inquiries can be directed to the corresponding authors.
