# Supplementary material for: Predicting tigecycline-related adverse events in infected patients: a machine learning approach with clinical interpretability
Source: Front Pharmacol. 2025 Nov 18;16:1697929. doi: 10.3389/fphar.2025.1697929 (PMC12669143; doi:10.3389/fphar.2025.1697929)
Supplement: Supplementary file 1 [file DataSheet1.pdf]

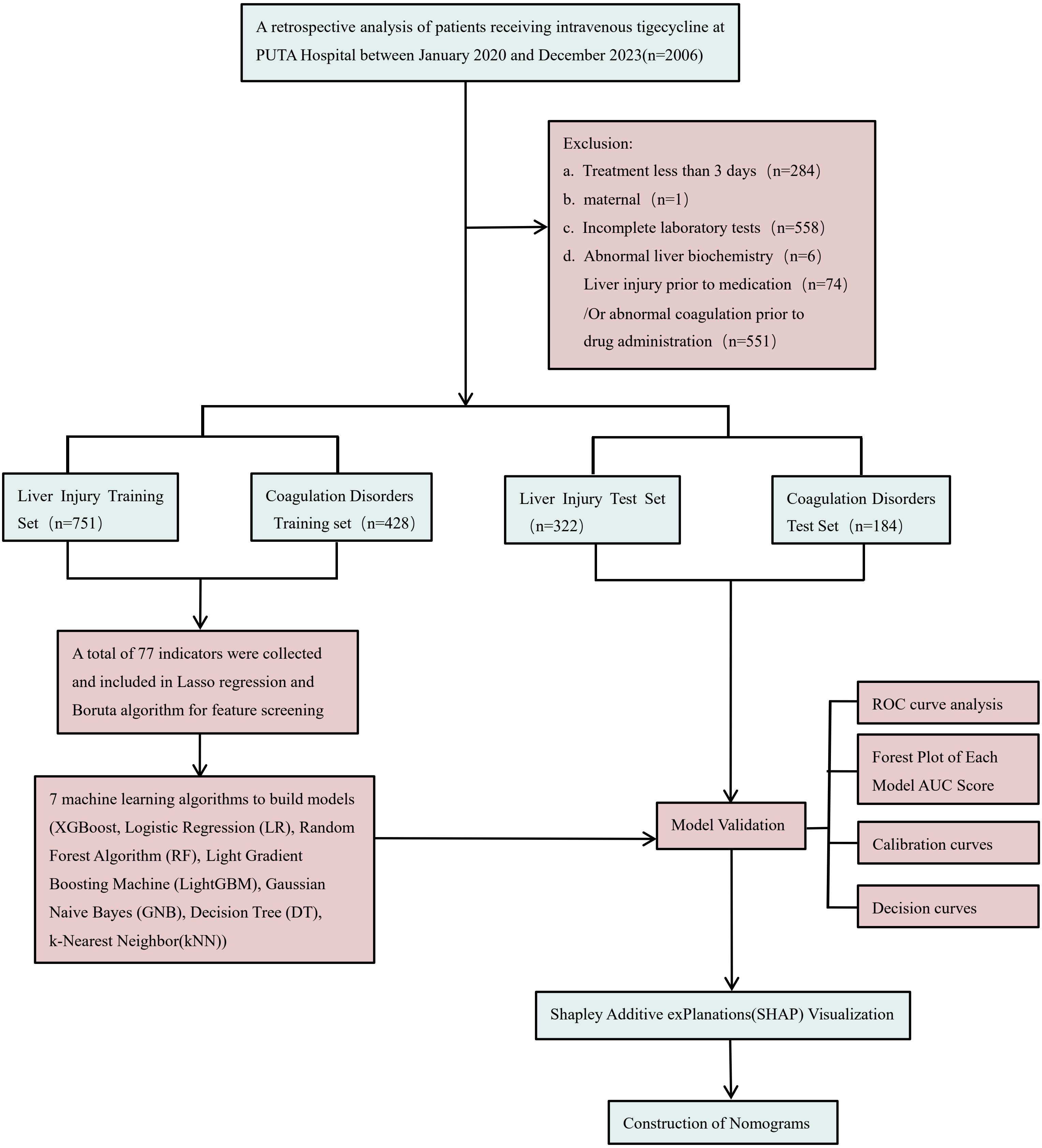

Supplementary Figure 1 Patient inclusion and exclusion flowchart

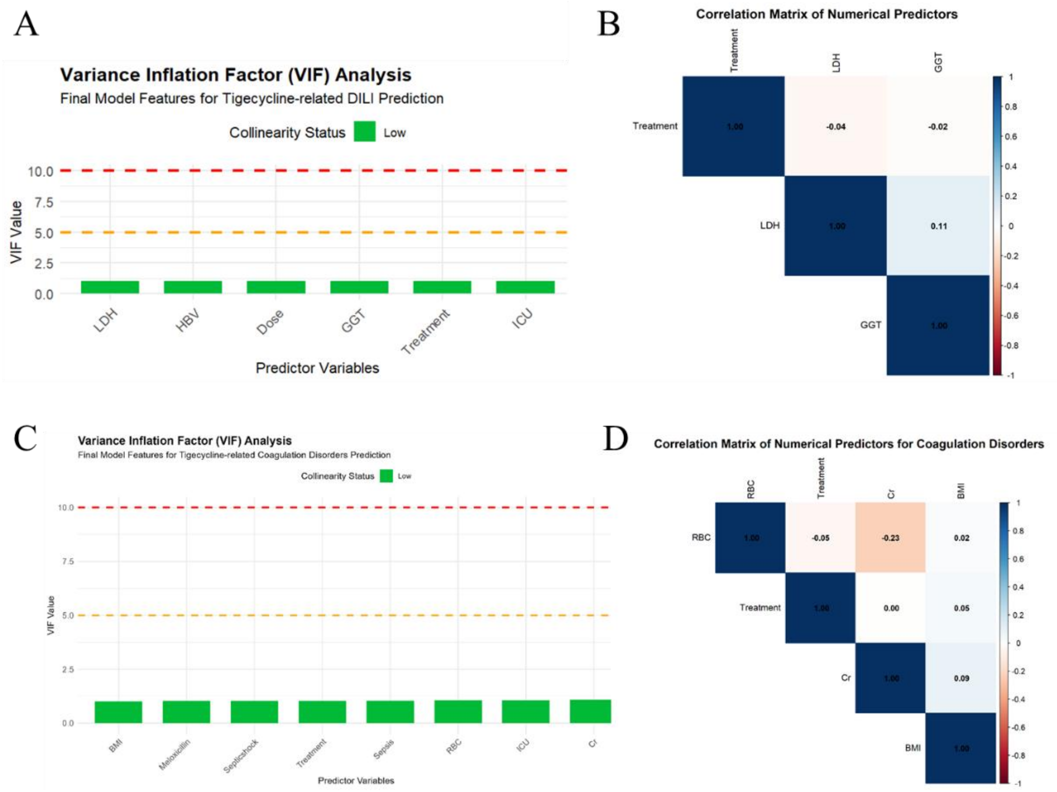

Supplementary Figure 2 VIF Analysis for Liver Injury and Coagulation Disorders Prediction. (A) VIF Analysis for Liver Injury; (B) Correlation Matrix of Numerical Predictors for Liver Injury; (C) VIF Analysis for Coagulation Disorders; (D) Correlation Matrix of Numerical Predictors for Coagulation Disorders.

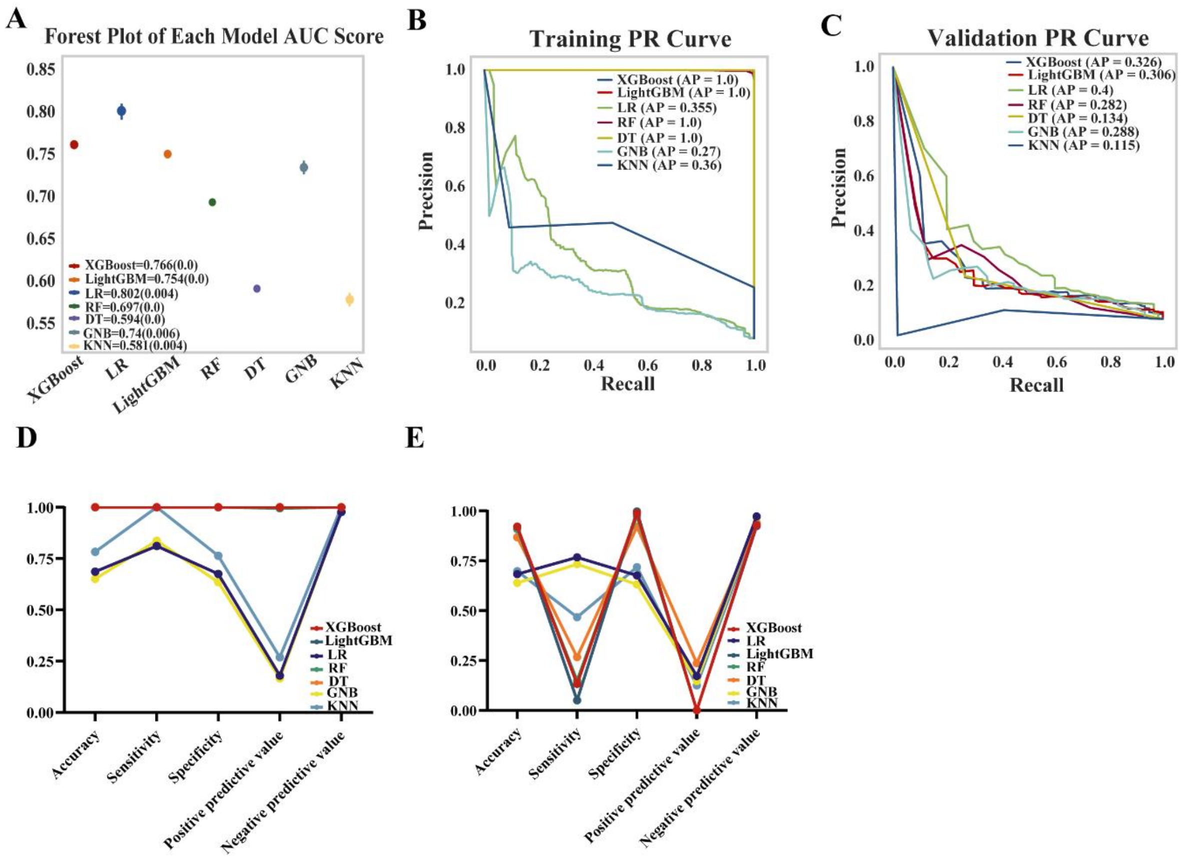

Supplementary Figure 3 Development, Performance Comparison, and Interpretability Analysis of ML Models for Liver Injury Prediction. (A) Forest plot of AUC values; (B) Training set PR curve; (C) Validation set PR curve; (D) Evaluation metrics for training set; (E) Evaluation metrics for Validation set.

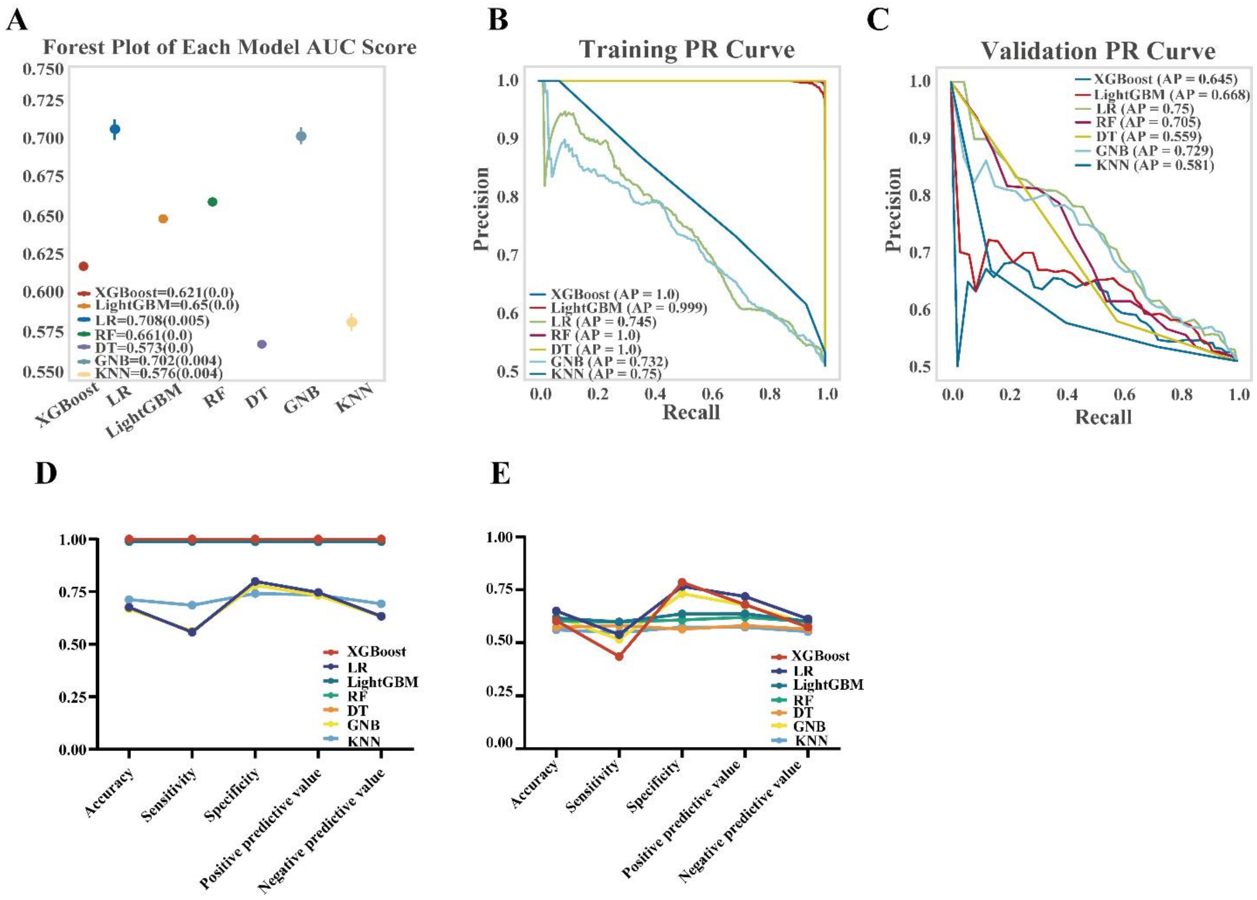

Supplementary Figure 4 Development, Performance Comparison, and Interpretability Analysis of ML Models for Predicting TGC-Induced Coagulation Disorders (A) Forest plot of AUC values; (B) Training set PR curve; (C) Validation set PR curve; (D) Evaluation metrics for training set; (E) Evaluation metrics for Validation set.

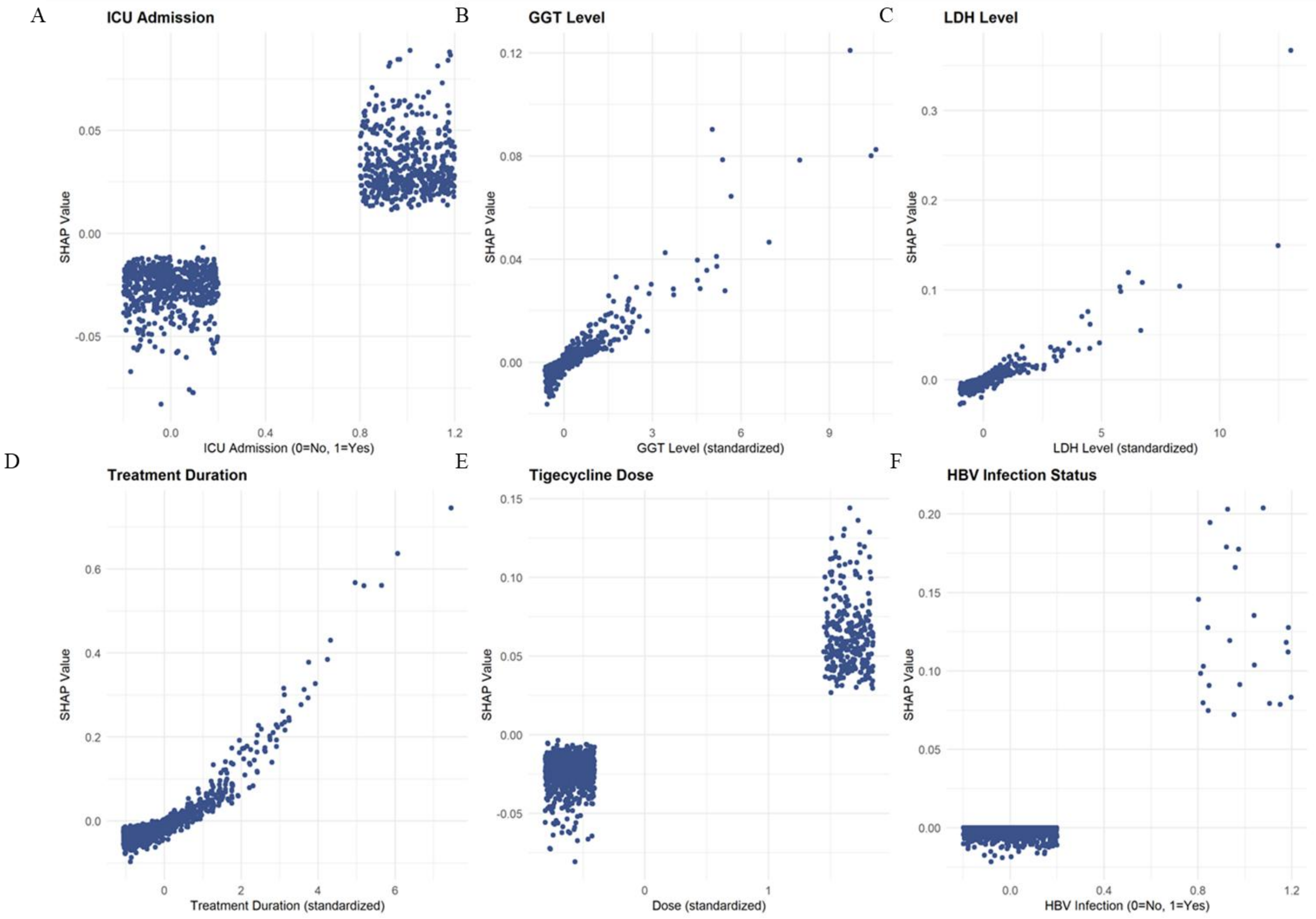

Supplementary Figure 5 Predicting Drug-Induced Liver Injury: SHAP Dependence of Key Predictors (e.g., ICU Admission, GGT)

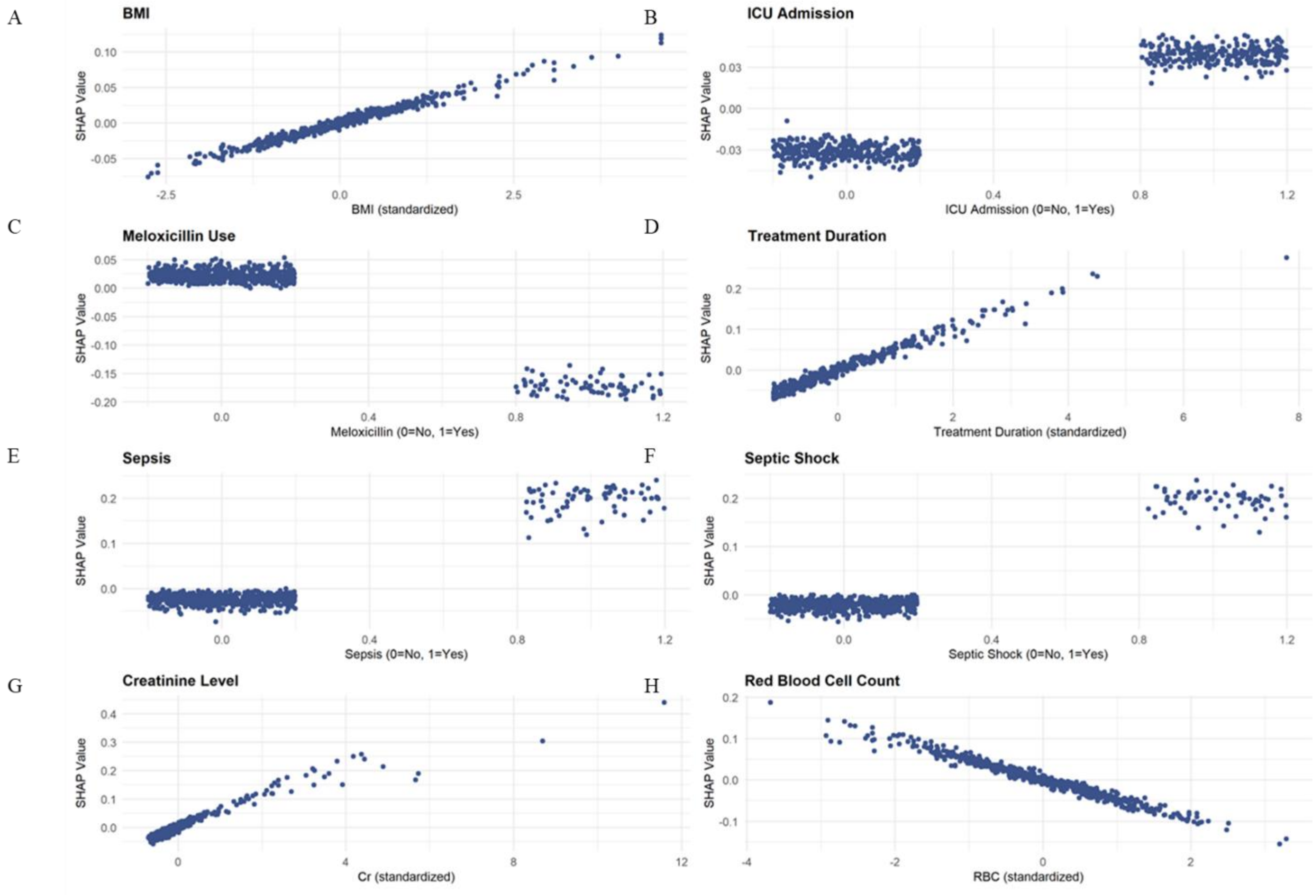

Supplementary Figure 6 Predicting Coagulation Dysfunction: SHAP Dependence of Key Clinical Features (e.g., BMI, Meloxicillin)

Table 1 The RUCAM scale

	Hepatocyte type		Cholestasis or mixed type		Evaluation
1、Time from medication to onset					
Not relevant	Reactions occur before starting the drug or more than 15 days after stopping		Reactions occur before starting the drug or more than 30 days after stopping the drug		correlation
Unknown	Can't get time on medication until onset		Can't get time on medication until onset		correlation
	Initial treatment	Subsequent treatment	Initial treatment	Subsequent treatment	Scoring
Start with the medicine					
Tip	5-90 days	1-15 days	5-90 days	1-90 days	+2
Suspicious	<5 days or >90days	>15 days	<5 days or >90 days	>90 days	+1
Start by going off the drugs					
Suspicious	≤15 days	≤15 days	≤30 days	≤30 days	+1
2、Course	ALT Peak versus upper limit of normal		The difference between ALP or TBIL peaks and the upper limit of normal		
post-discontinuation					
High Alert	> 50% reduction in 8 days		N/A		+3
Tip	⩾50% reduction within 30 days		⩾50% decrease in 180 days		+2
Suspicious	Not applicable after 30 days		A decrease of < 50% within 180 days		+1
No conclusion	No relevant data or decline ⩾50% after 30 days		Unchanged, rising, or no data		0
The opposite of a drug	Decrease < 50% or increase again after 30 days		N/A		-2
If the drug is still in use					
No conclusion	Everything		Everything		0
3、Risk factors	Alcohol		Alcohol or pregnancy		
Yes					+1
No					0
Age ≥55 years					+1
Age < 55 years					0
4、Concomitant medication					
The time of non-concomitant drug use does not coincide with the onset time					0
The time of concomitant drug use is consistent with the onset time					-1
It is known that concomitant drugs have hepatotoxicity and the use time is consistent with the onset time					-2
Evidence of concomitant liver injury (pharmaceutical response or valuable test)					-3
5、Among other reasons					
(1)recent infection with anti-HBc-IgM or anti-HCV or			All causes, including (1) and (2) , were		+2
evidence of other non-a or non-b hepatitis infections: biliary			completely excluded		+1
obstruction (ultrasound) ; Alcohol abuse AST/AIT ⩾2) a			All reasons in (1) were excluded		0
history of hypotension, shock, or hepatic ischemia within the			4-5 reasons in (1) were excluded		-2
last 2 weeks.			Fewer than 4 reasons in (1) were excluded		
(2) significant disease complications: clinical and/or laboratory			High suspicion for non-pharmaceutical		-3

indications of CMV, EBV, or herpesvirus infection.	factors		

	Hepatocyte type	Cholestasis or mixed type	Evaluation
6、Reports of previous drug-induced liver injury			
Reports of hepatotoxicity are included in the product description			+2
It's in the literature, but in the product			+1
No hepatotoxicity has been reported			0
7、Pharmaceutical reactions			
Positive	ALT elevation ⩾2 ULN with this agent alone	ALT or TBIL elevation ⩾ 2ULN with this agent alone	+3
Suspicious	ALT ⩾2 ULN after administration with concomitant drugs for the first occurrence of liver injury	ALT or TBIL elevation ⩾2 ULN when administered with a coadministration for the first occurrence of liver injury	+1
Negative	ALT is still in the normal range with the same medication	ALT or TBIL is still in the normal range	-2
Not done or not judged	Other conditions	Other conditions	0

Table 2 Missing Data Summary for Liver Injury Cohort Variables

Column	Missing Count	Missing Percentage
Age	0	0
Sex	0	0
BMI	0	0
Hypertension	0	0
Diabetes	0	0
CHD	0	0
MT	0	0
Drink	0	0
Smoke	0	0
ICU	0	0
MAV	0	0
Cefoperazone sulbactam	0	0
Meropenem	0	0
Biapenem	0	0
Polymyxin b	0	0
Piperacillin sulbactam	0	0
Meloxicillin	0	0
Dose	0	0
Treatment	0	0
Lung	0	0
Coeliac cavity	0	0
Skin	0	0
Blood	0	0
Urinary tract	0	0
Acinetobacter baumannii	0	0
Klebsiella pneumoniae	0	0
Candida albicans	0	0
E. coli	0	0
Enterococcus faecalis	0	0
Staphylococcus aureus	0	0
Hepatic cyst	0	0
Liver cirrhosis	0	0
Cholecystitis	0	0
Gallstone	0	0
HBV	0	0
Sepsis	0	0
Septic shock	0	0
ALT	0	0
AST	0	0
WBC	0	0
IBIL	0	0

Column	Missing Count	Missing Percentage
TBIL	0	0
ALP	0	0
TP	0	0
LDH	0	0
Cr	0	0
UA	0	0
BUN	0	0
GGT	0	0
CK	0	0
PLT	0	0
RBC	0	0
ApoA	0	0
ApoB	0	0
HDL	11	1.025163094
LDL	11	1.025163094
DD	0	0
Na	0	0
K	0	0
CL	0	0
Ca	0	0
FIB	0	0
PT	0	0
APTT	0	0
PT1	0	0
PCT	0	0
IL6	0	0
P	0	0
LYM	0	0
MONO	0	0
TG	0	0
NE	0	0
INR	0	0
TT	0	0
DBIL	0	0
HGB	0	0
ALB	76	7.082945014

Table 3 Missing Data Summary for Coagulation Disorders Cohort Variables

Column	Missing Count	Missing Percentage
Age	0	0
Sex	0	0
BMI	0	0
Hypertension	0	0
Diabetes	0	0
CHD	0	0
MT	0	0
Drink	0	0
Smoke	0	0
ICU	0	0
MAV	0	0
Cefoperazone sulbactam	0	0
Meropenem	0	0
Biapenem	0	0
Polymyxin b	0	0
Piperacillin sulbactam	0	0
Meloxicillinsodium sulbactam	0	0
Dose	0	0
Treatment	0	0
Lung	0	0
Coeliac cavity	0	0
Skin	0	0
Blood	0	0
Urinary tract	0	0
Acinetobacter baumannii	0	0
Klebsiella pneumoniae	0	0
Candida albicans	0	0
E coli	0	0
Enterococcus faecalis	0	0
Staphylococcus aureus	0	0
Hepatic cyst	0	0
Liver cirrhosis	0	0
Cholecystitis	0	0
Gallstone	0	0
HBV	0	0
Sepsis	0	0
Septic shock	0	0
ALT	0	0
AST	0	0
WBC	0	0
IBIL	0	0

Column	Missing Count	Missing Percentage
TBIL	0	0
ALP	0	0
TP	0	0
LDH	0	0
Cr	0	0
UA	0	0
BUN	0	0
GGT	0	0
CK	0	0
PLT	0	0
RBC	0	0
ApoA	0	0
ApoB	0	0
HDL	1	0.163398693
LDL	1	0.163398693
D.D	0	0
Na	0	0
K	0	0
CL	0	0
Ca	0	0
FIB	0	0
PT	0	0
APTT	0	0
PT.	0	0
PCT	0	0
IL.6	0	0
P	0	0
TG	0	0
LYM	0	0
NE	0	0
MONO	0	0
DBIL	0	0
Hb	0	0
INR	0	0
TT	0	0
ALB	56	9.150326797

Table 4 Description of the study variables

SN	Predictors	Description	Types	Values
1	Age	Age of the patient (years)	Continuous	25-101
2	BMI	Body mass index (kg/m^2)	Continuous	12.40-40.09
3	Treatment/d	Duration of drug treatment	Continuous	2.92-54.52
4	ICU	Patient admitted to ICU for treatment	Categorical	0 No ICU admission<br>1 With ICU admission
5	MAV	Mechanical assisted ventilation	Categorical	0 No Mechanical assisted<br>1 With Mechanical assisted
6	Gender	Sex of the patient	Categorical	1 male<br>2 female
7	Dose	Drug maintenance dose	Categorical	0 Maintenance dose≤50 mg<br>1 Maintenance dose≥100 mg
8	Hypertension	High blood pressure	Categorical	0 No history of hypertension<br>1 History of hypertension
9	Diabetes	History of diabetes	Categorical	0 No history of diabetes<br>1 History of diabetes
10	CHD	Coronary atherosclerotic heart disease	Categorical	0 No history of CHD<br>1 History of CHD
11	MT	Malignant tumour	Categorical	0 No history of MT<br>1 History of MT
12	Drink	History of alcohol consumption	Categorical	0 No history of drinking<br>1 History of drinking
13	Smoke	History of smoking	Categorical	0 No history of smoking<br>1 History of smoking
14	Cefoperazone sulbactam	Antibiotics	Categorical	0 No co-medication<br>1 With co-medication
15	Meropenem	Antibiotics	Categorical	0 No co-medication<br>1 With co-medication
16	Biapenem	Antibiotics	Categorical	0 No co-medication<br>1 With co-medication
17	Polymyxin b	Antibiotics	Categorical	0 No co-medication<br>1 With co-medication
18	Piperacillin sulbactam	Antibiotics	Categorical	0 No co-medication<br>1 With co-medication
19	Meloxicillin sodium sulbactam	Antibiotics	Categorical	0 No co-medication<br>1 With co-medication
20	Lung	Location of infection	Categorical	0 No an infection in the area<br>1 With an infection in the area
21	Abdominal cavity	Location of infection	Categorical	0 No an infection in the area<br>1 With an infection in the area
22	Skin	Location of infection	Categorical	0 No an infection in the area<br>1 With an infection in the area

SN	Predictors	Description	Types	Values
23	Blood	Bloodstream infection	Categorical	0 No an infection in the area<br>1 With an infection in the area
24	Urinary tract	Location of infection	Categorical	0 No an infection in the area<br>1 With an infection in the area
25	Sepsis	Infectious disease	Categorical	0 No history of Sepsis<br>1 History of Sepsis
26	Septic shock	Infectious disease	Categorical	0 No history of Septic shock<br>1 History of Septic shock
27	Acinetobacter baumannii	Pathogenic microorganism	Categorical	0 No infection with pathogens<br>1 Infected with pathogens
28	Klebsiella pneumoniae	Pathogenic microorganism.	Categorical	0 No infection with pathogens<br>1 Infected with pathogens
29	Candida albicans	Pathogenic microorganism	Categorical	0 No infection with pathogens<br>1 Infected with pathogens
30	Enterococcus faecalis	Pathogenic microorganism	Categorical	0 No infection with pathogens<br>1 Infected with pathogens
31	E. coli	Pathogenic microorganism	Categorical	0 No infection with pathogens<br>1 Infected with pathogens
32	Staphylococcus aureus	Pathogenic microorganism	Categorical	0 No infection with pathogens<br>1 Infected with pathogens
33	Hepatic cyst	Basic Liver Diseases	Categorical	0 No history of Hepatic cyst<br>1 History of Hepatic cyst
34	Liver cirrhosis	Basic Liver Diseases	Categorical	0 No history of Liver cirrhosis<br>1 History of Liver cirrhosis
35	Cholecystitis	Basic Liver Diseases	Categorical	0 No history of Cholecystitis<br>1 History of Cholecystitis
36	Gallstone	Basic Liver Diseases	Categorical	0 No history of Gallstone<br>1 History of Gallstone
37	HBV	Hepatitis B virus	Categorical	0 No history of HBV<br>1 History of HBV
38	ALT	Alanine Aminotransferase	Continuous	0.2-236.6
39	AST	AspartateTransaminase	Continuous	1.8-2521.8
40	ALP	Alkaline Phosphatase	Continuous	20.5-1301
41	ALB	Albumin	Continuous	0.2-76.6
42	GGT	γ -glutamyl transpeptidase	Continuous	6-1083.2
43	TBIL	Total bilirubin	Continuous	0.9-664.3
44	IBIL	Indirect bilirubin	Continuous	0.1-232.1
45	DBIL	Direct bilirubin	Continuous	0.1-432.2
46	TP	Total protein	Continuous	13.5-117.8
47	Cr	Creatinine	Continuous	20-1373.4
48	UA	Uric acid	Continuous	46-1165.8
49	BUN	Blood urea nitrogen	Continuous	0.9-56.61
50	CK	Creatine kinase	Continuous	4.5-8984

SN	Predictors	Description	Types	Values
51	ApoA	Apoprotein A	Continuous	0.02-2.06
52	ApoB	Apoprotein B	Continuous	0.01-1.68
53	HDL	High-density lipoprotein	Continuous	0.05-2.58
54	LDL	Low-density lipoprotein	Continuous	0.1-9.90
55	WBC	White blood cell	Continuous	0.1-128.69
56	LDH	Lactate dehydrogenase	Continuous	18.7-4233
67	PLT	Platelet	Continuous	1-866
68	RBC	Red blood cell	Continuous	0.81-6.63
69	D-D	D-D dimer	Continuous	0.06-80.00
60	FIB	Fibrinogen	Continuous	0.28-13.49
61	PT	Prothrombin Time	Continuous	8.9-89.4
62	APTT	Activated partial thromboplastin time	Continuous	17.1-180.0
63	PCT	Calcitonin	Continuous	0.01-100
64	IL-6	Interleukin-6	Continuous	1.5-5000
65	PT%	Prothrombin Time	Continuous	8.2-147
66	TG	Triglyceride	Continuous	0.07-29.3
67	LYM	Lymphocyte	Continuous	0-100.25
68	NE	Norepinephrine	Continuous	0.01-45.27
69	MONO/%	Monocyte count	Continuous	0-63.66
70	HGB	Haemoglobin	Continuous	4.7-185
71	INR	international normalized ratioinr	Continuous	0.76-8.61
72	TT	Thrombin time	Continuous	11.50-240
73	Na	Sodium	Continuous	98.7-208
74	K	Potassium	Continuous	1.9-9.07
75	Cl	Chlorine	Continuous	64.4-143.5
76	Ca	Calcium	Continuous	1.27-4.09
77	P	Phosphorus	Continuous	0.07-3.49

Table 5 Baseline Characteristics of the Training and Test Sets in the Liver Injury Risk Sample

Variables	Total (n=1073)	Train set (n=751)	Test set (n=322)	P
Age	71.00 (62.00, 79.00)	71.00 (62.00, 79.00)	71.00 (62.00, 79.00)	0.602
BMI（kg/m^2）	21.79 (19.72, 23.44)	21.80 (19.77, 23.48)	21.73 (19.58, 23.42)	0.721
Treatment/day	7.88 (5.05, 11.81)	7.86 (5.02, 11.97)	7.93 (5.12, 11.68)	0.514
ICU	491 (45.76)	343 (45.67)	148 (45.96)	0.930
MAV	339 (31.59)	235 (31.29)	104 (32.30)	0.745
Gender[n（%）]				
Male	817 (76.14)	565 (75.23)	252 (78.26)	0.286
Female	256 (24.86)	186 (25.77)	70 (22.74)	
Dose[n（%）]				
≤100 mg/d	784 (73.07)	554 (73.77)	230 (71.43)	0.428
≥200 mg/d	289 (26.93)	197 (26.23)	92 (28.57)	
Complication[n（%）]				
Hypertension	381 (35.51)	260 (34.62)	121 (37.58)	0.354
Diabetes	252 (23.49)	172 (22.90)	80 (24.85)	0.492
CHD	119 (11.09)	84 (11.19)	35 (10.87)	0.880
MT	199 (18.55)	146 (19.44)	53 (16.46)	0.250
Histology[n（%）]				
Drinking	20 (1.86)	17 (2.26)	3 (0.93)	0.139
Smoking	53 (4.90)	39 (5.20)	14 (4.30)	0.558
Drug combination[n（%）]				
Cefoperazone sulbactam	343 (32.00)	233 (31.00)	110 (34.00)	0.313
Meropenem	325 (30.29)	230 (30.63)	95 (29.50)	0.714
Biapenem	70 (6.52)	53 (7.06)	17 (5.28)	0.280
Polymyxin b	82 (7.64)	56 (7.46)	26 (8.08)	0.727
Piperacillin sulbactam	175 (16.31)	117 (15.58)	58 (18.01)	0.323
Meloxicillin sodium sulbactam	105 (9.79)	79 (10.52)	26 (8.08)	0.217
Site and type of infection[n（%）]				
Lung	845 (78.75)	589 (78.43)	256 (79.50)	0.693
Abdominal cavity	132 (12.30)	91 (12.12)	41 (12.73)	0.778
Skin	49 (4.57)	36 (4.79)	13 (4.04)	0.587
Blood	22 (2.05)	14 (1.86)	8 (2.48)	0.511
Urethra	30 (2.80)	22 (2.93)	8 (2.48)	0.685
Sepsis	137 (12.78)	98 (13.07)	39 (12.11)	0.668
Septic shock	127 (11.84)	86 (11.45)	41 (12.73)	0.551
Bacterium[n（%）]				
Acinetobacter baumannii	680 (63.37)	477 (63.52)	203 (63.04)	0.883

Table 5 Baseline Characteristics of the Training and Test Sets in the Liver Injury Risk Sample (Continued)

Variables	Total (n=1073)	Train set (n=751)	Test set (n=322)	P
Klebsiella pneumoniae	373 (34.76)	274 (36.49)	99 (30.75)	0.070
Candida albicans	76 (7.08)	50 (6.66)	26 (8.08)	0.407
Enterococcus faecalis	34 (3.17)	24 (3.20)	10 (3.11)	0.938
E. coli	85 (7.92)	55 (7.32)	30 (9.32)	0.268
Staphylococcus aureus	70 (6.52)	49 (6.53)	21 (6.52)	0.999
Basic liver disease[n（%）]				
Hepatic cyst	64 (5.97)	38 (5.06)	26 (8.08)	0.056
Liver cirrhosis	15 (1.40)	12 (1.60)	3 (0.93)	0.394
Cholecystitis	21 (1.96)	14 (1.86)	7 (2.17)	0.737
Gallstone	92 (8.57)	60 (7.99)	32 (9.94)	0.296
HBV	24 (2.24)	19 (2.53)	5 (1.55)	0.321
ALT($U \cdot L^{-1}$)	16.10 (10.50, 29.00)	16.70 (10.50, 29.10)	15.40 (10.63, 27.38)	0.470
AST($U \cdot L^{-1}$)	23.80 (16.50, 36.80)	24.20 (16.50, 37.35)	22.70 (16.63, 34.48)	0.401
ALP($U \cdot L^{-1}$)	79.00 (62.60, 103.00)	79.00 (62.70, 104.10)	78.80 (62.60, 100.23)	0.806
ALB/$g \cdot L^{-1}$	32.40 (28.70, 36.30)	32.40 (28.90, 36.30)	32.40 (28.43, 36.28)	0.917
GGT($U \cdot L^{-1}$)	34.00 (22.00, 58.0)	34.00 (22.00, 58.25)	34.10 (21.00, 56.38)	0.979
TBIL/$\mu mol \cdot L^{-1}$	9.00 (6.10, 13.90)	9.00 (6.20, 13.90)	8.70 (6.03, 14.18)	0.412
IBIL/$\mu mol \cdot L^{-1}$	5.40 (3.60, 8.50)	5.50 (3.70, 8.50)	5.30 (3.50, 8.50)	0.686
DBIL/$\mu mol \cdot L^{-1}$	3.20 (1.90, 5.20)	3.20 (2.00, 5.40)	3.20 (1.80, 4.98)	0.324
TP/$g \cdot L^{-1}$	64.40 (58.20, 70.70)	64.70 (57.80, 70.95)	64.15 (58.73, 69.68)	0.332
Cr/$\mu mol \cdot L^{-1}$	72.00 (55.00, 103.00)	72.00 (55.00, 102.00)	73.90 (56.00, 104.50)	0.500
UA/$\mu mol \cdot L^{-1}$	284.00 (195.00, 395.00)	280.00 (193.00, 394.00)	289.80 (203.95, 402.18)	0.364
BUN/$mmol \cdot L^{-1}$	6.00 (4.20, 9.15)	5.84 (4.20, 9.01)	6.16 (4.29, 9.81)	0.472
CK($U \cdot L^{-1}$)	73.00 (41.00, 170.00)	74.70 (39.60, 172.00)	69.50 (43.00, 162.75)	0.854
ApoA/$g \cdot L^{-1}$	0.71 (0.53, 0.90)	0.71 (0.52, 0.90)	0.70 (0.54, 0.89)	0.943
ApoB/$g \cdot L^{-1}$	0.58 (0.39, 0.76)	0.58 (0.40, 0.75)	0.58 (0.39, 0.76)	0.957
HDL/$mmol \cdot L^{-1}$	0.92 (0.68, 1.21)	0.93 (0.67, 1.21)	0.89 (0.70, 1.17)	0.843
LDL/$mmol \cdot L^{-1}$	2.00 (1.50, 2.70)	2.00 (1.40, 2.70)	2.00 (1.50, 2.80)	0.434
WBC/$10^9 \cdot L^{-1}$	9.55 (6.88, 12.890)	9.46 (6.84, 12.82)	9.975 (7.08, 13.17)	0.365
LDH($U \cdot L^{-1}$)	243.60 (189.80, 329.00)	241.00 (189.00, 326.75)	248.15 (191.25, 348.50)	0.306
PLT/$10^9 \cdot L^{-1}$	198.00 (144.00, 268.00)	200.00 (146.00, 269.20)	197.00 (141.00, 263.00)	0.513
RBC/$10^{12} \cdot L^{-1}$	3.80 (3.19, 4.35)	3.82 (3.18, 4.37)	3.77 (3.22, 4.27)	0.507
D-D/$mg \cdot L^{-1}$	2.08 (0.98, 4.98)	2.00 (0.95, 4.75)	2.24 (1.03, 5.25)	0.248
FIB/$g \cdot L^{-1}$	4.53 (3.20, 5.93)	4.62 (3.20, 6.04)	4.41 (3.20, 5.32)	0.166
PT/s	12.00 (11.00, 13.40)	12.10 (11.10, 13.40)	12.00 (10.90, 13.40)	0.248
APTT/s	30.10 (25.80, 36.97)	30.20 (25.70, 37.10)	30.00 (25.92, 35.40)	0.534
PCT/$ng \cdot mL^{-1}$	0.59 (0.19, 3.04)	0.59 (0.19, 3.18)	0.58 (0.18, 3.01)	0.515

Table 5 Baseline Characteristics of the Training and Test Sets in the Liver Injury Risk Sample (Continued)

Variables	Total (n=1073)	Train set (n=751)	Test set (n=322)	P
IL-6/pg·mL^{-1}	140.06 (65.66, 264.30)	141.46 (65.41, 273.57)	137.73 (67.92, 250.64)	0.550
PT%	89.00 (79.50, 98.60)	89.00 (79.50, 98.40)	90.00 (80.30, 99.15)	0.407
TG/mmol·L^{-1}	1.01 (0.72, 1.44)	0.99 (0.72, 1.41)	1.06 (0.73, 1.48)	0.321
LY/10^{9}·L^{-1}	0.80 (0.47, 1.25)	0.80 (0.49, 1.27)	0.78 (0.46, 1.21)	0.642
NE/10^{9}·L^{-1}	8.71 (5.98, 11.76)	8.72 (5.97, 11.72)	8.67 (6.01, 11.84)	0.693
MONO/%	0.47 (0.28, 0.71)	0.47 (0.29, 0.70)	0.48 (0.26, 0.74)	0.781
HGB/10^{9}·L^{-1}	113.00 (91.00, 131.00)	114.00 (92.00, 132.00)	113.00 (90.00, 128.00)	0.504
INR	1.05 (0.95, 1.16)	1.05 (0.96, 1.16)	1.05 (0.95, 1.16)	0.306
TT/s	17.90 (16.50, 19.20)	17.80 (16.40, 19.20)	17.90 (16.60, 19.20)	0.800
Na/mmol·L^{-1}	135.30 (131.00, 139.10)	135.20 (131.00, 139.00)	135.50 (131.55, 139.25)	0.362
K/mmol·L^{-1}	3.73 (3.38, 4.14)	3.75 (3.40, 4.18)	3.70 (3.36, 4.07)	0.113
Cl/ mmol·L^{-1}	100.20 (96.00, 104.80)	100.10 (95.90, 104.90)	100.60 (96.05, 104.68)	0.513
Ca/mmol·L^{-1}	2.13 (2.01, 2.25)	2.13 (2.02, 2.25)	2.13 (2.00, 2.25)	0.620
P/mmol·L^{-1}	0.97 (0.75, 1.20)	0.97 (0.75, 1.21)	0.98 (0.76, 1.19)	0.929

Table 6 Baseline Characteristics of the Training and Test Sets in the Coagulopathy Risk Sample

Variables	Total (n=612)	Train set (n=428)	Test set (n=184)	P
Age	71.00 (63.00, 79.00)	71.00 (63.00, 79.00)	71.50 (63.75, 79.00)	0.805
BMI（kg/m^2）	21.69 (19.73, 23.20)	21.80 (19.85, 23.09)	21.58 (19.49, 23.55)	0.651
Treatment/day	7.99 (5.11, 11.97)	7.99 (5.09, 11.91)	8.00 (5.14 ,12.05)	0.974
ICU	266 (43.46)	190 (44.39)	76 (41.30)	0.480
MAV	171 (27.94)	119 (27.80)	52 (28.26)	0.446
Gender[n（%）]				
Male	473 (77.29)	327 (76.40)	146 (79.35)	0.425
Female	139 (22.71)	101 (23.60)	38 (20.65)	
Dose[n（%）]				
≤100 mg/d	444 (72.55)	306 (71.50)	138 (75.00)	0.373
≥200 mg/d	168 (27.45)	122 (28.50)	46 (25.00)	
Complication[n（%）]				
Hypertension	228 (37.25)	162 (37.85)	66 (35.87)	0.642
Diabetes	144 (23.53)	102 (23.83)	42 (22.83)	0.788
CHD	72 (11.76)	53 (12.38)	19 (10.33)	0.469
MT	110 (17.97)	77 (17.99)	33 (17.93)	0.987
Histology[n（%）]				
Drinking	13 (2.12)	8 (1.87)	5 (2.72)	0.718
Smoking	35 (5.72)	26 (6.07)	9 (4.89)	0.563
Drug combination[n（%）]				
Cefoperazone sulbactam	197 (32.19)	135 (31.54)	62 (33.70)	0.601
Meropenem	171 (27.94)	119 (27.80)	52 (28.26)	0.908
Biapenem	38 (6.21)	26 (6.07)	12 (6.52)	0.834
Polymyxin b	44 (7.19)	32 (7.48)	12 (6.52)	0.675
Piperacillin sulbactam	98 (16.01)	67 (15.65)	31 (16.85)	0.712
Meloxicillin sodium	70 (11.44)	48 (11.21)	22 (11.96)	0.792
Site and type of infection[n（%）]				
Lung	498 (81.37)	344 (80.37)	154 (83.70)	0.333
Abdominal cavity	68 (11.11)	46 (10.75)	22 (11.96)	0.663
Skin	19 (3.10)	16 (3.74)	3 (1.63)	0.168
Blood	9 (1.47)	7 (1.64)	2 (1.09)	0.880
Urethra	13 (2.12)	7 (1.64)	6 (3.26)	0.331
Sepsis	69 (11.27)	54 (12.62)	15 (8.15)	0.109
Septic shock	60 (9.80)	45 (10.51)	15 (8.15)	0.368
Bacterium[n（%）]				
Acinetobacter baumannii	391 (63.89)	280 (65.42)	111 (60.33)	0.229
Klebsiella pneumoniae	230 (37.58)	160 (37.38)	70 (38.04)	0.877
Candida albicans	44 (7.19)	28 (6.54)	16 (8.70)	0.344
Enterococcus faecalis	19 (3.10)	16 (3.74)	3 (1.63)	0.168

Table 6 Baseline Characteristics of the Training and Test Sets in the Coagulopathy Risk Sample

Variables	Total (n=612)	Train set (n=428)	Test set (n=184)	P
E. coli	42 (6.86)	31 (7.24)	11 (5.98)	0.570
Staphylococcus aureus	30 (4.90)	17 (3.97)	13 (7.07)	0.104
Basic liver disease[n（%）]				
Hepatic cyst	37 (6.05)	28 (6.54)	9 (4.89)	0.432
Liver cirrhosis	2 (0.33)	2 (0.47)	0 (0.00)	>0.999
Cholecystitis	13 (2.12)	10 (2.34)	3 (1.63)	0.803
Gallstone	57 (9.31)	42 (9.81)	15 (8.15)	0.517
HBV	12 (1.96)	10 (2.34)	2 (1.09)	0.481
ALT(U·L^{-1})	15.30 (10.10, 27.90)	16.30 (10.70, 27.90)	14.35 (9.08, 26.63)	0.109
AST(U·L^{-1})	22.55 (16.38, 32.83)	22.50 (16.40, 32.25)	22.65 (16.30, 33.63)	0.930
ALP(U·L^{-1})	80.00 (65.00, 101.93)	80.00 (65.00, 103.10)	80.00 (62.35, 100.00)	0.863
ALB/g·L^{-1}	33.00 (29.80, 37.625)	33.20 (30.20, 37.65)	32.80 (29.10, 37.33)	0.315
GGT(U·L^{-1})	33.85 (21.98, 60.18)	34.10 (22.00, 61.03)	31.20 (21.88, 54.50)	0.675
TBIL/μmol·L^{-1}	8.70 (6.00, 12.90)	8.30 (5.90, 12.43)	9.25 (6.50, 14.15)	0.059
IBIL/μmol·L^{-1}	5.30 (3.60, 8.13)	5.20 (3.60, 8.03)	5.50 (3.68, 8.28)	0.322
DBIL/μmol·L^{-1}	2.90 (1.80, 4.70)	2.80 (1.80, 4.60)	3.25 (1.90, 5.40)	0.053
TP/g·L^{-1}	65.50 (60.28, 71.70)	65.45 (60.58, 72.00)	65.60 (59.20, 71.05)	0.675
Cr/μmol·L^{-1}	69.90 (54.83, 92.25)	69.00 (54.00, 93.25)	71.75 (55.15, 90.00)	0.914
UA/μmol·L^{-1}	287.00 (196.00, 377.70)	283.50 (195.15, 389.00)	290.50 (197.90, 367.25)	0.604
BUN/mmol·L^{-1}	5.69 (4.100, 7.85)	5.70 (4.16, 7.85)	5.64 (3.95, 7.85)	0.479
CK(U·L$^{-1)}$	71.10 (42.00, 161.03)	71.10 (42.00, 166.25)	71.50 (40.75, 156.75)	0.713
ApoA/g·L^{-1}	0.75 (0.58, 0.93)	0.75 (0.58, 0.95)	0.73 (0.58, 0.91)	0.415
ApoB/g·L^{-1}	0.61 (0.43,0.80)	0.63 (0.45, 0.80)	0.58 (0.38, 0.78)	0.091
HDL/mmol·L^{-1}	0.98 (0.73, 1.30)	0.99 (0.74, 1.29)	0.93 (0.72, 1.35)	0.635
LDL/mmol·L^{-1}	2.30 (1.70, 2.80)	2.30 (1.70, 2.90)	2.10 (1.60, 2.70)	0.081
WBC/10^{9}·L^{-1}	9.67 (7.14, 12.68)	9.58 (7.10, 12.66)	9.93 (7.18, 12.82)	0.688
LDH(U·L^{-1})	235.55 (184.15, 313.25)	233.50 (183.00, 311.10)	240.65 (188.73, 317.45)	0.417
PLT/10^{9}·L^{-1}	215.00 (178.00 ,265.25)	216.50 (178.00, 266.00)	212.00 (178.75 ,264.25)	0.908
RBC/10^{12}·L^{-1}	3.98 (3.42, 4.43)	3.98 (3.45, 4.44)	4.00 (3.35, 4.42)	0.881
D-D/mg·L^{-1}	1.76 (0.84, 4.15)	1.68 (0.80, 4.03)	2.05 (0.95, 4.22)	0.177
FIB/g·L^{-1}	4.68 (3.37, 6.04)	4.64 (3.33, 5.95)	4.79 (3.46, 6.21)	0.329
PT/s	11.60 (10.70, 12.30)	11.50 (10.70, 12.30)	11.60 (10.70, 12.30)	0.439
APTT/s	28.15 (24.20 ,32.50)	28.00 (24.08, 32.43)	28.60 (24.70, 32.75)	0.157
PCT/ng·mL^{-1}	0.49 (0.17, 2.44)	0.47 (0.16, 2.43)	0.52 (0.20, 2.53)	0.188
IL-6/pg·mL^{-1}	136.67 (66.59, 225.57)	136.67 (70.89, 225.59)	132.24 (53.52 ,224.42)	0.523
PT%	92.90 (85.30, 101.70)	92.90 (85.30, 101.70)	92.90 (86.20, 100.60)	0.630
TG/mmol·L^{-1}	0.99 (0.72, 1.38)	0.99 (0.74, 1.37)	0.99 (0.70, 1.45)	0.839
LY/10^{9}·L^{-1}	0.84 (0.51, 1.26)	0.85 (0.51, 1.27)	0.78 (0.49, 1.22)	0.282
NE/10^{9}·L^{-1}	8.60 (6.24, 11.53)	8.815 (6.39, 11.76)	8.03 (5.81, 10.53)	0.075

Table 6 Baseline Characteristics of the Training and Test Sets in the Coagulopathy Risk Sample

Variables	Total (n=612)	Train set (n=428)	Test set (n=184)	P
MONO/%	0.50 (0.31, 0.73)	0.49 (0.30, 0.71)	0.51 (0.32, 0.79)	0.287
HGB/$10^9 \cdot L^{-1}$	119.00 (99.75, 135.00)	120.00 (101.00, 134.25)	119.00 (97.00, 135.00)	0.664
INR	1.00 (0.93, 1.07)	1.00 (0.93, 1.07)	1.01 (0.93, 1.06)	0.600
TT/s	17.60 (16.30, 18.90)	17.60 (16.30, 19.00)	17.60 (16.20, 18.73)	0.577
Na/mmol·L^{-1}	135.35 (131.28, 138.90)	135.80 (131.60, 139.00)	134.30 (130.40, 138.55)	0.057
K/mmol·L^{-1}	3.72 (3.38, 4.12)	3.73 (3.37, 4.13)	3.71 (3.39, 4.10)	0.938
Cl/mmol/ L^{-1}	99.70 (96.00, 104.00)	100.00 (96.00, 104.20)	99.55 (96.18, 103.80)	0.620
Ca/mmol·L^{-1}	2.15 (2.04, 2.27)	2.15 (2.04, 2.27)	2.14 (2.04, 2.27)	0.466
P/mmol·L^{-1}	0.98 (0.75, 1.18)	0.97 (0.75, 1.18)	0.99 (0.79, 1.17)	0.598

Table 7 Occurrence of liver injury in patients treated with TGC

projects	Number of cases	Percentage (%)	95% confidence interval
Meets DILI diagnostic criteria	99	9.14	7.40-10.9
RUCAM score ≥6	89	8.22	6.60-9.90
RUCAM score <6	10	0.92	0.40-1.50
Liver function tests			
TBIL≥1×ULN	302	27.9	25.2-30.6
TBIL≥2×ULN	107	9.88	8.10-11.7
DBIL≥1×ULN	385	35.5	32.7-38.4
DBIL≥2×ULN	198	18.3	16.0-20.6
IBIL≥1×ULN	104	9.60	7.80-11.4
IBIL≥2×ULN	24	2.22	1.30-3.10
ALT≥1×ULN	178	16.4	14.2-18.6
ALT≥2×ULN	56	5.20	3.90-6.60
AST≥1×ULN	334	31.1	28.4-33.9
AST≥2×ULN	113	10.5	8.70-12.4
ALP≥1×ULN	467	43.1	40.2-46.1
ALP≥2×ULN	96	8.86	7.20-10.6

Table 8 Univariate analysis of the TGC-induced liver injury training set

Variables	Total (n=751)	Control group(n=691)	Liver injury group(n=60)	χ^2/t/z	P
Age	71.0 (62. 0, 79.0)	71.0 (62.5, 79.0）	69.5 (57.5, 80.0）	-1.06	0.288
BMI（kg/m^2）	21.80 (19.77, 23.48)	21.78 (19.73, 23.45)	22.41 (20.66, 23.55)	-1.43	0.154
Treatment/day	7.86 (5.02, 11.97)	7.38 (4.99, 11.03)	12.08 (8.17, 18.87)	-5.89	<.001
ICU	343 (45.67)	299 (43.27)	44 (73.33)	20.11	<.001
MAV	235 (31.29)	201 (29.09)	34 (56.67)	19.53	<.001
Gender[n（%）]					
Male	565 (75.23)	518 (74.96)	47 (78.33)	0.34	0.562
Female	186 (24.77)	173 (25.04)	13 (21.67)		
Dose[n（%）]					
≤100 mg/d	554 (73.77)	526 (76.12)	28 (46.67)	24.75	<.001
≥200 mg/d	197 (26.23)	165 (23.88)	32 (53.33)		
Complication[n（%）]					
Hypertension	260 (34.62)	235 (34.01)	25 (41.67)	1.43	0.232
Diabetes	172 (22.90)	155 (22.43)	17 (28.33)	1.09	0.297
CHD	84 (11.19)	76 (11.00)	8 (13.33)	0.30	0.582
MT	146 (19.44)	131 (18.96)	15 (25.00)	1.29	0.257
Histology[n（%）]					
Drinking	17 (2.26)	14 (2.03)	3 (5.00)	1.07	0.302
Smoking	39 (5.19)	36 (5.21)	3 (5.00)	0.00	1.000
Drug combination[n（%）]					
Cefoperazone sulbactam	233 (31.03)	220 (31.84)	13 (21.67)	2.67	0.102
Meropenem	230 (30.63)	208 (30.10)	22 (36.67)	1.12	0.290
Biapenem	53 (7.06)	48 (6.95)	5 (8.33)	0.02	0.889
Polymyxin b	56 (7.46)	46 (6.66)	10 (16.67)	6.63	0.010
Piperacillin sulbactam	117 (15.58)	104 (15.05)	13 (21.67)	1.84	0.175
Meloxicillin sodium sulbactam	79 (10.52)	76 (11.00)	3 (5.00)	2.11	0.146
Site and type of infection[n（%）]					
Lung	589 (78.43)	540 (78.15)	49 (81.67)	0.40	0.525
Abdominal cavity	91 (12.12)	83 (12.01)	8 (13.33)	0.09	0.763
Skin	36 (4.79)	34 (4.92)	2 (3.33)	0.06	0.813
Blood	14 (1.86)	12 (1.74)	2 (3.33)	0.14	0.704
Urethra	22 (2.93)	21 (3.04)	1 (1.67)	0.04	0.837
Sepsis	98 (13.07)	87 (12.59)	11 (18.64)	1.75	0.185
Septic shock	86 (11.45)	74 (10.71)	12 (20.00)	4.70	0.030
Bacterium[n（%）]					
Acinetobacter baumannii	477 (63.52)	445 (64.40)	32 (53.33)	2.92	0.088
Klebsiella pneumoniae	274 (36.48)	248 (35.89)	26 (43.33)	1.32	0.251
Candida albicans	50 (6.66)	44 (6.37)	6 (10.00)	0.66	0.416

Table 8 Univariate analysis of the TGC-induced liver injury training set. (Continued)

Variables	Total (n=751)	Control group(n=691)	Liver injury group(n=60)	χ^2/t/z	P
Enterococcus faecalis	24 (3.20)	22 (3.18)	2 (3.33)	0.00	1.000
E. coli	55 (7.32)	51 (7.38)	4 (6.67)	0.00	1.000
Staphylococcus aureus	49 (6.52)	45 (6.51)	4 (6.67)	0.00	1.000
Basic liver disease[n（%）]					
Hepatic cyst	38 (5.06)	37 (5.35)	1 (1.67)	0.89	0.346
Liver cirrhosis	12 (1.60)	12 (1.74)	0 (0.00)	-	0.613
Cholecystitis	14 (1.86)	13 (1.88)	1 (1.67)	0.00	1.000
Gallstone	60 (7.99)	54 (7.81)	6 (10.00)	0.12	0.726
HBV	19 (2.53)	15 (2.17)	4 (6.67)	2.89	0.089
ALT($U \cdot L^{-1}$)	16.70 (10.50, 29.10)	16.70 (10.50, 29.40)	15.95 (10.38, 26.70)	-0.45	0.651
AST($U \cdot L^{-1}$)	24.20 (16.50, 37.35)	24.00 (16.50, 37.35)	26.50 (17.48, 36.17)	-0.66	0.512
ALP($U \cdot L^{-1}$)	79.00 (62.70, 104.10)	79.00 (62.25, 102.25)	80.00 (65.75, 113.50)	-0.88	0.380
ALB/$g \cdot L^{-1}$	32.40 (28.90, 36.30)	32.50 (29.00, 36.30)	30.65 (27.30, 34.00)	-2.03	0.043
GGT($U \cdot L^{-1}$)	34.00 (22.00, 58.25)	34.00 (22.00, 57.25)	41.50 (23.75, 79.25)	-1.33	0.184
TBIL/$\mu mol \cdot L^{-1}$	9.00 (6.20, 13.90)	9.00 (6.10, 13.80)	9.95 (7.30, 14.90)	-1.56	0.118
IBIL/$\mu mol \cdot L^{-1}$	5.50 (3.70, 8.50)	5.50 (3.70, 8.50)	6.00 (4.38, 8.50)	-1.16	0.245
DBIL/$\mu mol \cdot L^{-1}$	3.20 (2.00, 5.40)	3.20 (2.00, 5.40)	3.35 (2.15, 5.58)	-1.09	0.275
TP/$g \cdot L^{-1}$	64.70 (57.80, 70.95)	65.00 (58.45, 71.25)	61.05 (55.45, 66.38)	-2.82	0.005
Cr/$\mu mol \cdot L^{-1}$	72.00 (55.00, 102.00)	71.40 (54.00, 101.50)	77.60 (56.50, 118.22)	-1.14	0.256
UA/$\mu mol \cdot L^{-1}$	280.00 (193.00, 394.00)	279.70 (193.00, 393.10)	295.00 (193.85, 396.75)	-0.16	0.705
BUN/$mmol \cdot L^{-1}$	6.00 (4.20, 9.15)	5.92 (4.20, 9.10)	6.30 (3.92, 10.10)	-0.34	0.871
CK ($U \cdot L^{-1}$)	74.70 (39.60, 172.00)	73.00 (39.00, 166.50)	100.90 (44.50, 260.25)	-1.65	0.100
ApoA/$g \cdot L^{-1}$	0.71 (0.52, 0.90)	0.71 (0.53, 0.90)	0.71 (0.49, 0.91)	-0.41	0.685
ApoB/$g \cdot L^{-1}$	0.58 (0.40, 0.75)	0.58 (0.40, 0.75)	0.62 (0.36, 0.80)	-0.72	0.473
HDL/$mmol \cdot L^{-1}$	0.93 (0.67, 1.21)	0.93 (0.67, 1.23)	0.93 (0.68, 1.08)	-0.97	0.334
LDL/$mmol \cdot L^{-1}$	2.00 (1.40, 2.70)	2.00 (1.50, 2.70)	2.00 (1.20, 2.52)	-1.47	0.141
WBC/$10^9 \cdot L^{-1}$	9.46 (6.83, 12.82)	9.43 (6.77, 12.75)	9.80 (8.21, 13.95)	-1.34	0.179
LDH ($U \cdot L^{-1}$)	241.00 (189.00, 326.75)	240.00 (187.95, 319.50)	275.65 (198.00, 435.25)	-2.23	0.026
PLT/$10^9 \cdot L^{-1}$	200.00 (146.00, 269.20)	200.00 (146.00, 269.20)	188.50 (146.00, 272.50)	-0.39	0.697
RBC/$10^{12} \cdot L^{-1}$	3.82 (3.17, 4.37)	3.83 (3.19, 4.37)	3.79 (3.08, 4.27)	-0.92	0.358
D-D/$mg \cdot L^{-1}$	2.00 (0.95, 4.75)	1.94 (0.91, 4.69)	2.59 (1.11, 5.32)	-1.64	0.101
FIB/$g \cdot L^{-1}$	4.62 (3.20, 6.04)	4.64 (3.24, 6.21)	4.30 (3.14, 5.62)	-0.84	0.401
PT/s	12.10 (11.10, 13.40)	12.10 (11.10, 13.40)	12.10 (11.10, 13.40)	-0.22	0.823
APTT/s	30.20 (25.70, 37.10)	30.30 (25.70, 36.94)	29.95 (25.62, 40.23)	-0.11	0.911
PCT/$ng \cdot mL^{-1}$	0.59 (0.19, 3.18)	0.56 (0.19, 3.21)	0.94 (0.29, 2.69)	-1.21	0.225
IL-6/$pg \cdot mL^{-1}$	141.46 (65.41, 273.57)	142.66 (65.58, 270.23)	133.80 (56.34, 353.09)	-0.41	0.685
PT%	89.00 (79.50, 98.40)	89.00 (79.50, 98.60)	87.10 (77.00, 96.60)	-0.50	0.617
TG/$mmol \cdot L^{-1}$	0.99 (0.72, 1.41)	0.98 (0.72, 1.38)	1.15 (0.77, 1.63)	-1.82	0.069

Table 8 Univariate analysis of the TGC-induced liver injury training set. (Continued)

Variables	Total (n=751)	Control group(n=691)	Liver injury group(n=60)	χ^2/t/z	P
LY/$10^9 \cdot L^{-1}$	0.80 (0.49, 1.27)	0.81 (0.49, 1.26)	0.78 (0.47, 1.36)	-0.12	0.901
NE/$10^9 \cdot L^{-1}$	8.72 (5.97, 11.72)	8.63 (5.96, 11.61)	10.16 (6.97, 13.93)	-2.02	0.043
MONO/%	0.47 (0.29, 0.70)	0.47 (0.29, 0.70)	0.41 (0.24, 0.68)	-0.98	0.330
HGB/$10^9 \cdot L^{-1}$	114.00 (92.00, 132.00)	114.00 (92.00, 132.00)	109.50 (92.75, 129.75)	-0.34	0.732
INR	1.05 (0.96, 1.16)	1.05 (0.96, 1.16)	1.05 (0.96, 1.17)	-0.16	0.874
TT/s	17.80 (16.40, 19.20)	17.80 (16.40, 19.20)	18.15 (16.58, 19.85)	-1.06	0.289
Na/mmol$\cdot L^{-1}$	135.20 (131.00, 139.00)	135.10 (131.05, 139.00)	135.95 (129.00, 140.35)	-0.25	0.801
K/mmol$\cdot L^{-1}$	3.75 (3.40, 4.17)	3.76 (3.40, 4.16)	3.71 (3.40, 4.38)	-0.01	0.990
Cl/mmol$\cdot L^{-1}$	100.10 (95.90, 104.90)	100.10 (95.90, 104.60)	101.20 (96.30, 106.03)	-0.71	0.477
Ca/mmol$\cdot L^{-1}$	2.13 (2.01, 2.25)	2.13 (2.02, 2.25)	2.05 (1.97, 2.26)	-2.05	0.040
P/mmol$\cdot L^{-1}$	0.97 (0.75, 1.21)	0.96 (0.75, 1.21)	1.02 (0.75, 1.17)	-0.14	0.887

Table 9 Variance inflation factor (VIF) analysis results for Liver Injury.

Variable	VIF Collinearity Status	Interpretation
Treatment	1.032	Low No multicollinearity
LDH	1.014	Low No multicollinearity
GGT	1.03	Low No multicollinearity
ICU	1.033	Low No multicollinearity
Dose	1.021	Low No multicollinearity
HBV	1.015	Low No multicollinearity

Table 10 Risk factors and their parameters of the logistic model.

Variables	β	S.E	Z	*P*	OR (95%CI)
(Intercept)	-3.706	0.307	-12.085	0.0	0.025 (0.013-0.043)
HBV	1.306	0.654	1.998	0.046	3.691 (0.91-12.412)
ICU	0.929	0.323	2.877	0.004	2.532 (1.366-4.883)
Dose	1.196	0.296	4.039	<.001	3.305 (1.852-5.938)
LDH	0.125	0.114	1.102	0.271	1.134 (0.899-1.407)
GGT	0.18	0.128	1.403	0.16	1.197 (0.898-1.513)
Treatment	0.64	0.116	5.505	<.001	1.896 (1.516-2.395)

OR: Odds Ratio, CI: Confidence Interval

Table 11 Univariate analysis of the TGC-induced coagulopathy training set.

Variables	Total (n=428)	Control group (n=209)	Coagulation disorders group (n=219)	χ^2/t/z	P
Age	71.00 (63.00, 79.00)	70.00 (63.00, 79.00)	72.00 (64.00, 80.00)	-1.60	0.109
BMI（kg/m2）	21.79 (19.85, 23.09)	21.32 (18.83, 22.86)	21.98 (20.43, 23.64)	-3.12	0.002
Treatment/day	7.99 (5.08, 11.91)	7.91 (5.03, 11.29)	8.00 (5.21, 12.46)	-1.21	0.225
ICU	190 (44.39)	71 (33.97)	119 (54.34)	17.97	<.001
MAV	127 (29.67)	40 (19.14)	87 (39.73)	21.72	<.001
Gender[n（%）]					
Male	327 (76.40)	156 (74.64)	171 (78.08)	0.70	0.402
Female	101 (23.60)	53 (25.36)	48 (21.92)		
Dose[n（%）]					
≤100 mg/d	306 (71.50)	157 (75.12)	149 (68.04)	2.63	0.105
≥200 mg/d	122 (28.50)	52 (24.88)	70 (31.96)		
Complication[n（%）]					
Hypertension	162 (37.85)	70 (33.49)	92 (42.01)	3.30	0.069
Diabetes	102 (23.83)	49 (23.44)	53 (24.20)	0.03	0.854
CHD	53 (12.38)	18 (8.61)	35 (15.98)	5.35	0.021
MT	77 (17.99)	34 (16.27)	43 (19.63)	0.82	0.365
Histology[n（%）]					
Drinking	8 (1.87)	5 (2.39)	3 (1.37)	0.18	0.672
Smoking	26 (6.07)	15 (7.18)	11 (5.02)	0.87	0.351
Drug combination[n（%）]					
Cefoperazone sulbactam	135 (31.54)	73 (34.93)	62 (28.31)	2.17	0.141
Meropenem	119 (27.80)	43 (20.57)	76 (34.70)	10.64	0.001
Biapenem	26 (6.07)	13 (6.22)	13 (5.94)	0.02	0.902
Polymyxin b	32 (7.48)	11 (5.26)	21 (9.59)	2.89	0.089
Piperacillin sulbactam	67 (15.65)	40 (19.14)	27 (12.33)	3.76	0.053
Meloxicillin sodium sulbactam	48 (11.21)	36 (17.22)	12 (5.48)	14.82	<.001
Site and type of infection[n（%）]					
Lung	344 (80.37)	166 (79.43)	178 (81.28)	0.23	0.630
Abdominal cavity	46 (10.75)	22 (10.53)	24 (10.96)	0.02	0.885
Skin	16 (3.74)	5 (2.39)	11 (5.02)	2.06	0.152
Blood	7 (1.64)	4 (1.91)	3 (1.37)	0.00	0.950
Urethra	7 (1.64)	4 (1.91)	3 (1.37)	0.00	0.950
Sepsis	54 (12.62)	11 (5.26)	43 (19.63)	20.03	<.001
Septic shock	45 (10.51)	11 (5.26)	34 (15.53)	11.97	<.001
Bacterium[n（%）]					
Acinetobacter baumannii	280 (65.42)	142 (67.94)	138 (63.01)	1.15	0.284
Klebsiella pneumoniae	160 (37.38)	77 (36.84)	83 (37.90)	0.05	0.821

Table 11 Univariate analysis of the TGC-induced coagulopathy training set. (Continued)

Variables	Total (n=428)	Control group (n=209)	Coagulation disorders group (n=219)	χ^2/t/z	P
Candida albicans	28 (6.54)	15 (7.18)	13 (5.94)	0.27	0.604
Enterococcus faecalis	16 (3.74)	7 (3.35)	9 (4.11)	0.17	0.679
E. coli	31 (7.24)	12 (5.74)	19 (8.68)	1.37	0.242
Staphylococcus aureus	17 (3.97)	8 (3.83)	9 (4.11)	0.02	0.881
Basic liver disease[n（%）]					
Hepatic cyst	28 (6.54)	15 (7.18)	13 (5.94)	0.27	0.604
Liver cirrhosis	2 (0.47)	0 (0.00)	2 (0.91)	-	0.499
Cholecystitis	10 (2.34)	5 (2.39)	5 (2.28)	0.00	1.000
Gallstone	42 (9.81)	14 (6.70)	28 (12.79)	4.48	0.034
HBV	10 (2.34)	5 (2.39)	5 (2.28)	0.00	1.000
ALT($U \cdot L^{-1}$)	16.30 (10.70, 27.90)	15.40 (10.10, 27.90)	16.70 (11.05, 27.90)	-0.45	0.652
AST($U \cdot L^{-1}$)	22.50 (16.40, 32.25)	21.60 (16.40, 31.70)	23.50 (16.40, 33.35)	-0.61	0.544
ALP($U \cdot L^{-1}$)	80.00 (65.00, 103.10)	80.30 (68.10, 99.00)	78.00 (62.85, 105.00)	-0.58	0.565
ALB/$g \cdot L^{-1}$	33.20 (30.20, 37.65)	34.22 (31.00, 38.60)	32.50 (29.20, 36.90)	-2.70	0.007
GGT($U \cdot L^{-1}$)	34.10 (22.00, 61.02)	34.20 (22.00, 65.00)	34.00 (21.65, 57.75)	-0.39	0.700
TBIL/$\mu mol \cdot L^{-1}$	8.30 (5.90, 12.43)	8.30 (6.20, 12.60)	8.60 (5.90, 12.25)	-0.28	0.776
IBIL/$\mu mol \cdot L^{-1}$	5.20 (3.60, 8.03)	5.20 (3.60, 8.40)	5.20 (3.60, 7.80)	-0.69	0.492
DBIL/$\mu mol \cdot L^{-1}$	2.80 (1.80, 4.60)	2.60 (1.70, 4.70)	3.10 (1.90, 4.55)	-1.00	0.319
TP/$g \cdot L^{-1}$	65.45 (60.58, 72.00)	66.60 (61.90, 72.30)	64.70 (59.40, 70.75)	-2.54	0.011
Cr/$\mu mol \cdot L^{-1}$	69.00 (54.00, 93.25)	67.00 (52.00, 85.10)	73.00 (56.50, 112.00)	-2.71	0.007
UA/$\mu mol \cdot L^{-1}$	283.50 (195.15, 389.00)	290.50(203.00, 370.80)	280.00 (186.00, 409.60)	-0.00	0.999
BUN/$mmol \cdot L^{-1}$	5.70 (4.16, 7.85)	5.40 (4.10, 7.16)	5.94 (4.20, 9.05)	-2.86	0.004
CK($U \cdot L^{-1}$)	71.10 (42.00, 166.25)	62.70 (42.00, 127.00)	83.00 (44.50, 195.95)	-2.42	0.016
ApoA/$g \cdot L^{-1}$	0.75 (0.58, 0.95)	0.75 (0.61, 0.93)	0.75 (0.56, 0.95)	-0.49	0.624
ApoB/$g \cdot L^{-1}$	0.62 (0.45, 0.80)	0.63 (0.47, 0.80)	0.62 (0.45, 0.81)	-0.22	0.824
HDL/$mmol \cdot L^{-1}$	0.99 (0.74, 1.29)	1.01 (0.72, 1.30)	0.96 (0.74, 1.26)	-0.33	0.744
LDL/$mmol \cdot L^{-1}$	2.30 (1.70, 2.90)	2.40 (1.80, 2.90)	2.20 (1.60, 2.90)	-1.91	0.057
WBC/$10^9 \cdot L^{-1}$	9.58 (7.10, 12.66)	9.22 (7.16, 12.04)	10.05 (6.97, 13.01)	-1.32	0.187
LDH($U \cdot L^{-1}$)	233.50 (183.00, 311.10)	228.00(180.00, 298.00)	236.10 (186.10, 320.60)	-1.15	0.249
PLT/$10^9 \cdot L^{-1}$	216.50 (178.00, 266.00)	222.40(189.00, 268.00)	208.00 (169.50, 260.00)	-2.30	0.022
RBC/$10^{12} \cdot L^{-1}$	3.98 (3.45, 4.44)	4.09 (3.57, 4.57)	3.86 (3.29, 4.32)	-3.23	0.001
D-D/$mg \cdot L^{-1}$	1.67 (0.80, 4.03)	1.19 (0.68, 3.28)	2.14 (1.07, 4.54)	-3.90	<.001
FIB/$g \cdot L^{-1}$	4.64 (3.33, 5.95)	4.49 (3.28, 5.67)	4.72 (3.46, 6.21)	-1.07	0.284
PT/s	11.50 (10.70, 12.30)	11.40 (10.60, 12.30)	11.60 (10.80, 12.30)	-1.39	0.163
APTT/s	28.00 (24.08, 32.42)	27.20 (23.60, 31.40)	28.50 (24.40, 33.10)	-2.39	0.017
PCT/$ng \cdot mL^{-1}$	0.46 (0.16, 2.42)	0.33 (0.13, 2.09)	0.59 (0.20, 2.97)	-2.64	0.008
IL-6/$pg \cdot mL^{-1}$	136.67 (70.89, 225.59)	133.70 (75.78, 204.76)	137.35 (69.96, 255.10)	-1.06	0.291
PT%	92.90 (85.30, 101.70)	94.00 (86.20, 101.90)	92.90 (85.30, 100.35)	-1.35	0.179

TG/mmol·L^{-1}	0.99 (0.74, 1.37)	0.98 (0.70, 1.39)	1.01 (0.76, 1.33)	-0.45	0.652

Table 11 Univariate analysis of the TGC-induced coagulopathy training set. (Continued)

Variables	Total (n=428)	Control group (n=209)	Coagulation disorders group (n=219)	χ^2/t/z	P
LY/10^9·L^{-1}	0.85 (0.51, 1.27)	0.91 (0.58, 1.34)	0.77 (0.47, 1.23)	-2.08	0.038
NE/10^9·L^{-1}	8.82 (6.39, 11.76)	8.50 (6.33, 11.09)	9.11 (6.57, 12.80)	-1.83	0.068
MONO/%	0.49 (0.30, 0.71)	0.50 (0.32, 0.70)	0.48 (0.30, 0.75)	-0.17	0.863
HGB/10^9·L^{-1}	120.00(101.00, 134.25)	123.00(102.00, 136.00)	115.00 (98.00, 132.00)	-2.31	0.021
INR	1.00 (0.93, 1.07)	0.99 (0.92, 1.07)	1.01 (0.94, 1.07)	-1.39	0.164
TT/s	17.60 (16.30, 19.00)	17.80 (16.40, 19.10)	17.40 (16.30, 19.00)	-1.07	0.283
Na/mmol·L^{-1}	135.80 (131.60, 139.00)	136.00(132.00, 139.30)	135.60 (131.10, 138.75)	-0.63	0.528
K/mmol·L^{-1}	3.73 (3.37, 4.13)	3.73 (3.37, 4.11)	3.71 (3.40, 4.14)	-0.16	0.870
Cl/mmol·L^{-1}	100.00 (96.00, 104.20)	99.30 (95.50, 103.20)	100.10 (96.40, 105.20)	-1.25	0.210
Ca/mmol·L^{-1}	2.15 (2.04, 2.27)	2.16 (2.06, 2.29)	2.14 (2.02, 2.25)	-2.26	0.024
P/mmol·L^{-1}	0.97 (0.75, 1.18)	0.98 (0.75, 1.16)	0.96 (0.76, 1.19)	-0.30	0.766

Table 12 Variance inflation factor (VIF) analysis results for Coagulation Disorders.

Variable	VIF Collinearity Status	Interpretation
Treatment	1.03	Low No multicollinearity
Meloxicillin	1.026	Low No multicollinearity
RBC	1.052	Low No multicollinearity
Cr	1.085	Low No multicollinearity
BMI	1.021	Low No multicollinearity
Sepsis	1.045	Low No multicollinearity
Septic shock	1.027	Low No multicollinearity
ICU	1.062	Low No multicollinearity

Table 13 Risk factors and their parameters of the logistic model.

Variables	β	S.E	Z	*P*	OR (95%CI)
(Intercept)	-0.241	0.152	-1.588	0.112	0.786(0.583-1.058)
BMI	0.235	0.112	2.109	0.035	1.266 (1.02-1.582)
Cr	0.257	0.153	1.681	0.093	1.293 (0.995-1.805)
RBC	-0.226	0.114	-1.988	0.047	0.798 (0.637-0.995)
Treatment	0.208	0.111	1.868	0.062	1.231 (0.993-1.539)
Sepsis	1.092	0.379	2.885	0.004	2.982 (1.459-6.52)
Infective shock	0.974	0.383	2.545	0.011	2.648 (1.283-5.826)
meloxicillin use	-1.099	0.377	-2.919	0.004	0.333 (0.153-0.679)
ICU	0.462	0.219	2.114	0.035	1.588 (1.034-2.44)

OR: Odds Ratio, CI: Confidence Interval
